# A Method for WD40 Repeat Detection and Secondary Structure Prediction

**DOI:** 10.1371/journal.pone.0065705

**Published:** 2013-06-11

**Authors:** Yang Wang, Fan Jiang, Zhu Zhuo, Xian-Hui Wu, Yun-Dong Wu

**Affiliations:** 1 Lab of Computational Chemistry and Drug Design, Laboratory of Chemical Genomics, Peking University Shenzhen Graduate School, Shenzhen, P. R. China; 2 College of Chemistry, Peking University, Beijing, P. R. China; Università di Padova, Italy

## Abstract

WD40-repeat proteins (WD40s), as one of the largest protein families in eukaryotes, play vital roles in assembling protein-protein/DNA/RNA complexes. WD40s fold into similar β-propeller structures despite diversified sequences. A program **WDSP** (WD40 repeat protein Structure Predictor) has been developed to accurately identify WD40 repeats and predict their secondary structures. The method is designed specifically for WD40 proteins by incorporating both local residue information and non-local family-specific structural features. It overcomes the problem of highly diversified protein sequences and variable loops. In addition, WDSP achieves a better prediction in identifying multiple WD40-domain proteins by taking the global combination of repeats into consideration. In secondary structure prediction, the average Q3 accuracy of WDSP in jack-knife test reaches 93.7%. A disease related protein LRRK2 was used as a representive example to demonstrate the structure prediction.

## Introduction

WD40-repeat domains/proteins, as one of largest protein families, mainly provide platforms to assemble proteins, DNA or RNA into functional complexes [1], [2]. These protein complexes play roles in DNA replication [3], [4], [5], transcription [6], RNA processing [7], histone modification [8]/recognition [9], protein degradation [10], [11] and other processes [12], [13], [14], [15].

**Figure 1:**
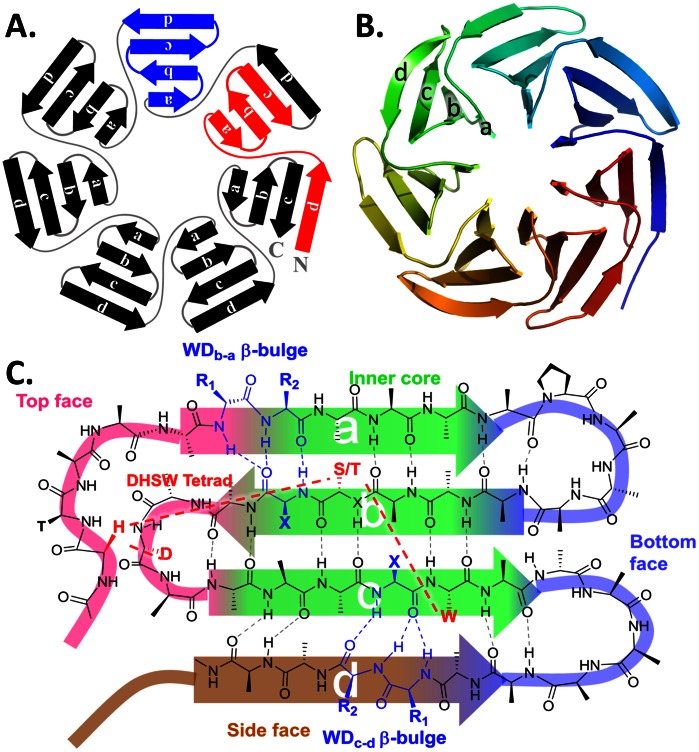
The structural hierarchy of WD40. **(A)** WD40 domain in 2D scheme. The definitions of WD40 blades and WD40 repeats are different. A WD40 blade, highlighted in blue, is S_a_-S_b_-S_c_-S_d_. A WD40 repeat, highlighted in red, is S_d_-S_a_-S_b_-S_c_. (**B**) The classical tertiary structure of a WD40 domain. (**C**) Topology and structural features of a WD blade. Top, bottom and side surfaces and inner core part are drawn in different colors. The residues and corresponding dashed lines highlighted in red are involved in DHSW tetrad hydrogen bonded network. The residues in blue are involved in β-bulges. Normally, two β-bulges (WD_b–a_ and WD_c–d_) exist in one WD40 blade.

A WD40 repeat usually contains 40–60 residues with conserved GH (Gly-His) near its N-terminus and conserved WD (Trp-Asp) at its C-terminus. As shown in **Figure 1A**, each such repeat folds into a 4-strand β-sheet. Sequentially, a WD40 repeat is composed of strands d(S_d_), a(S_a_), b(S_b_) and c(S_c_) in order but structurally S_a_, S_b_, S_c_ and S_d_, are aligned from inside to outside. Loops connecting the sequential strands are called loop ab (L_ab_), loop bc (L_bc_), loop cd (L_cd_) and loop da (L_da_) in the text. Typically, each WD40 domain contains 7 (the most common) to 8 repeats, which fold into an encircled 7/8-bladed β-propeller structure. In few cases, WD40 domains only contain 6 repeats. The 7^th^ repeat is provided by another protein to form an enclosed β-propeller, such as SEC13 [16] or SEH1 [17]. Some WD40-repeat proteins (WD40s) have as many as 14 repeats. Examples are SRO7 [18] and AIP1 [19], which fold into two enclosed β-propellers.

Due to their vital functions, a number of methods are available for detecting WD40 repeats from primary sequences. In 1994, Neer *et al* provided a regular expression for WD40 repeat identification [1], which was successfully applied to annotate 29 WD40s. In 2000, 32 functional subfamilies were further identified to contain WD40 repeats [20]. Currently, the most widely used methods, PROSITE [21], Pfam [22], SMART [23] and REP [24], are all providing annotation for WD40 repeats. Particularly, REP [24] is one of the default annotation methods used in UniProt Database.

However, the sequence diversity makes the identification of WD40 repeats difficult [1], [2], [13], [15]. In the Superfamily database [25], 1222 proteins in *Homo sapiens* have been annotated to contain 1305 WD40 domains. Their average pairwise sequence identity is only about 21%. The low sequence identity restricts the current methods from identifying the WD40 repeats completely. Even in reproducing WD40 domains with crystal structures, the average WD40 repeat number per domain ranges from 3.4 to 5.9. For example, DNA damage-binding protein 2 (DDB2) is a 7-repeat WD40 protein with solved crystal structure (PDB code: 3EI4) [26]. However, only 5 repeats are identified by UniProt, REP and SMART and 3 repeats are detected by PROSITE and Pfam. Moreover, the detected WD40 repeats are normally shorter than they really are in its crystal structure. The missed parts of sequence impede the accurate slicing of WD40 domain. In addition, these repeat detection methods cannot provide domain topology information without knowing the secondary structure in the repeat.

The topology of a WD40 domain can be established when its secondary structure is accurately predicted as well. Through over 50 years of development, the state-of-art secondary structure predicting methods have been improved dramatically [27]. The widely used methods, for example, GOR4 [28], PHD [29], PROF [30], SSpro [31] and PSIPRED [32], are able to provide reasonably good predictions. Especially for PSIPRED, the overall three-state accuracy (Q3) has reached 81.4% (±0.6%) [33]. However, accurate prediction of β-sheets remains a challenge compared with α-helixes, because β-sheets require hydrogen bonds between linearly distant residues. Some defects in the secondary structure prediction are usually observed in β-strand. The predicted β-strands are sometimes shorter, longer or shifted by several residues as compared with crystal structures. These defects lead to incorrect topology predictions, three-dimensional structure modeling and functional residue interpretation.

The low sequence identity does not impede WD40s on folding into the similar structure, β-propeller. According the previous studies, one possibility is that they share some vital structural features. As shown in **Figure 1C**, the conserved hydrogen-bonded DHSW tetrad, formed by Asp-His-Ser/Thr-Trp [34], [35], and β-bulges [36], [37], [38] are indispensable for maintaining protein stability [35] and provide binding ability [36]. Here, we present a fast, robust and accurate method, WD40-repeat protein Structure Predictor (**WDSP**), which incorporates local residue propensities, nonlocal information of structural features and repeat number preference to enhance the prediction.

Using this method, we are able to identify new WD40 repeats and domains from protein sequences. Over 2000 known WD40 repeats are identified in the Swiss-Prot database. In addition, the method also detects 76 novel WD40s in the database. For example, Tau 91 from *S. cerevisiae* was not detected to be a WD40 protein by the currently available methods, even though the crystal structure is available (PDB code 2J04). Finally, one disease related WD40 protein, LRRK2 [39], [40], [41], [42], [43], is used to demonstrate the capability of WD40 repeat annotation and secondary structure prediction.

## Materials and Methods

### The Overview of WDSP

**Figure 2:**
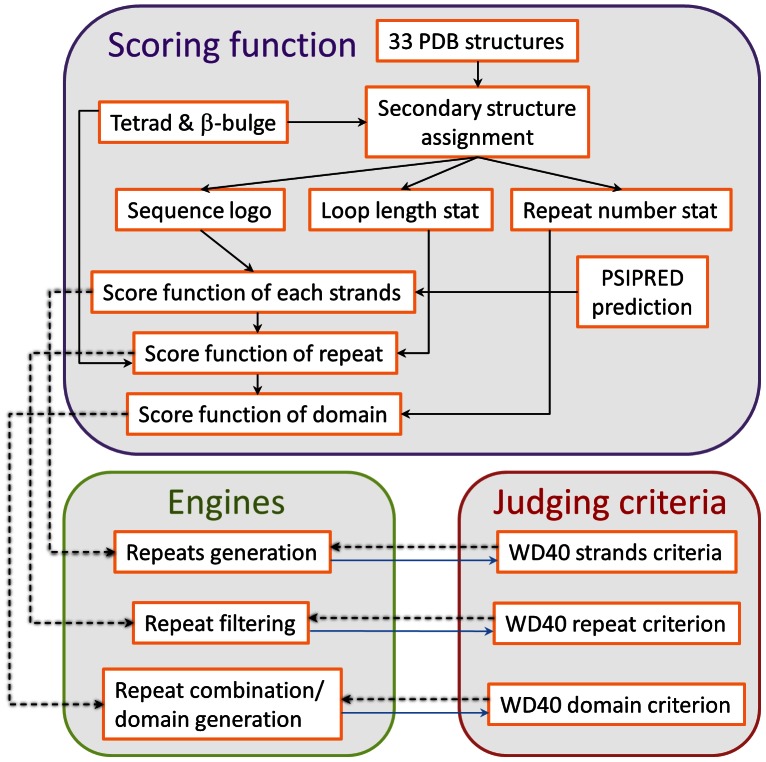
Development of the WDSP. The scoring functions, the searching/optimization engines and the evaluation criteria are developed independently. The scoring functions and criteria are used in the later optimization procedures (dashed arrows). The criteria values are optimized based on the results and the performances of the engines (blue solid arrows).

The WDSP method consists of three independent parts (**Figure 2**). The first part includes three scoring functions, which are used to comprehensively estimate the quality of predicted strands, repeats and domains. The second part is composed of multiple engines, which are able to remove the repeats with low scores and further combine optimal WD40 repeats into closure WD40 domains according to the scoring functions. The third part is the criteria that support the judgment of WD40 strands, repeats and domain.

### An Unbiased Data Set of Available WD40 Crystal Structures

The first step of scoring function development is to establish a database of WD40 proteins with known crystal structures, which are classified by both CATH/SCOP and assignments from the literature. Every currently known WD40 protein has at least one DHSW tetrad H-bond network. By calculating their WD40 domain pairwise sequence identities, 33 WD40 proteins were selected in the training set (**Table S1**). These proteins have no more than 32% pairwise sequence identities in the WD40 domains. 239 WD40 repeats in 33 proteins have average 16% pairwise sequence identity (93.3% of repeats have less than 30% pairwise sequence identity). This ensures a statistically unbiased training set.

### Assignment of Secondary Structure Elements

**Figure 3:**
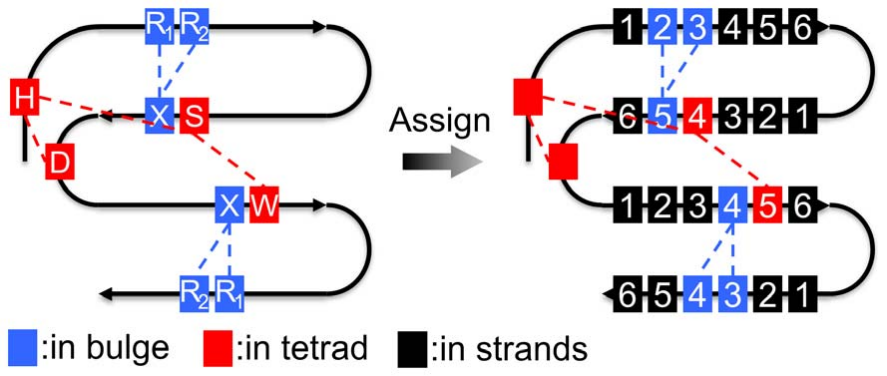
Secondary structure assignment of WD40 repeats based on the structural features. The residues in β-bulges and the DHSW tetrad are shown in blue and red colors, respectively. These residues are aligned in a higher priority. The blocks with numbers are assigned to be residues in the β-strand secondary structure.

The second step is to assign four strands (S_a_, S_b_, S_c_ and S_d_) and align the sequences according to their secondary structures. To avoid secondary structure assignment variation among different methods [44], we assign 239 WD40 repeats by using the structural features as “landmarks”. As shown in **Figure 3**, the R_1_ and R_2_ of the WD_b–a_ β-bulge were assigned as the 2^nd^ and the 3^rd^ residues of S_a_. The Ser/Thr residues in the tetrad and the X position in WD_b–a_ were the 4^th^ and 5^th^ residues of S_b_. The X positions of WD_c–d_ and Trp residue in the tetrad were assigned as the 4^th^ and 5^th^ residues of S_c_. The 3^rd^ and the 4^th^ residues of S_d_ are the R_1_ and R_2_ residues of WD_c–d_. As usual, the length of each strand is kept to be six residues [1], [2], [12], [15], [20]. Thus, the remaining residues in the β-strands can be assigned according to these landmarks. On average, the resulting assignment of 33 WD proteins has over 90% Q3 similarity compared with the assignment of DSSP [45] or STRIDE [46]. This value is similar to the intrinsic discrepancy among different assignment methods [47], [48].

### The Generation of WD40 Repeat Sequence Logo

**Figure 4:**
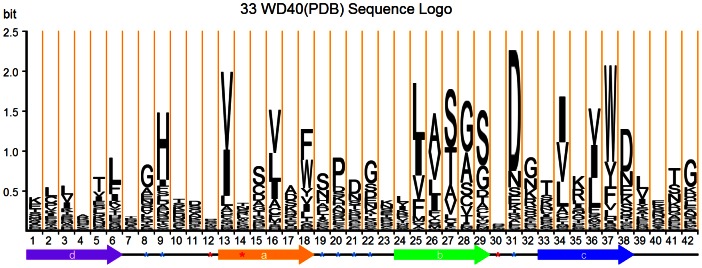
Sequence logo of the WD40 repeat in which the heights of letters show the conservations of the residues at the position. The total height of the letters represents the information entropy of the position. The secondary structure is depicted below. The positions highlighted by red asterisks are potential hotspots positions on the top face involved in the protein-protein interactions. The blue asterisks indicate the relatively conserved positions in the loops are included in the S_aa_ in equation (3). The detailed residue frequencies in the sequence logo are listed in Table S4.

In the secondary structure assignment, all six-residue β-strands were preferentially and exactly aligned. The left loop regions were aligned using the BLOSUM62 score matrix. For the loop region, we discard loops that are longer than 15 residues (less than 4% of the dataset). The remaining loops are then aligned by normal multiple sequence alignment. Figure 4 shows the sequence logo of the WD40 repeat derived from these alignments. This sequence logo has some unique features compared to currently known sequence logos and it will be discussed in detail in the results section.

In order to establish a reliable sequence logo, the potential fluctuation of amino acid frequencies needs to be excluded because the number of WD40 repeats with available crystal structures is limited. The consistency of the residue frequencies has been analyzed by dividing the dataset into two groups. One group contains 119 randomly chosen WD40 repeats and the remaining 120 WD40 repeats belong to the other group. Two sequence logos have been developed from each of the two groups. The similarity between two logos is then compared (**Figure S1**). This process was repeated 10 times with ten independent divisions of 239 WD40 repeats. The similarity coefficients of the ten pairs of logos are calculated. The similarity coefficient *S* between two distributions X = {*x_i_*}, Y = {*y_i_*} is calculated as:
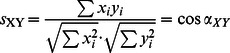
(1)


S_XY_ can be regarded as the value of cos(α). α is the angle between two 42×20 dimensional vectors X and Y (the sequence logo is composed of 42 residues in length with each of the 20 amino acids possible at every position), which represent two tested frequency logos. When and only when two distributions are the same after normalization, the similarity coefficient is S_XY_ = 1.

According to our structural-feature-based assignment, the average S value is **0.89**. The self-consistent test indicates the sequence logo is reliable for developing the scoring function.

### The Scoring Functions in WDSP

The scoring functions are applied to evaluate the probability of a sequence fragment to be a WD40 strand, a repeat or a domain. The score of a single WD40 repeat is composed of four terms: (a) the propensities of individual residues (*S_aa_*) at the different positions on every strand and one WD40 repeat; (b) the preferences for different loop lengths (*S_loop_len_*), (c) the existence of cooperative H-bonds within the DHSW tetrads (*S_corr_*) and (d) the secondary structure score as given by PSIPRED:
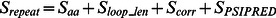
(2)


*S_aa_* is the weighted sum of the amino acid propensities(on a logarithmic scale) at the 31 positions within a WD40 repeat, which include the 6×4 positions in the well aligned strands and some relatively conserved position in the loops. As shown in **Figure 4**, these residues marked by blue asterisks are located at L_da_ (positions 8, 9), L_ab_ (positions 19, 20, 21, 22) and L_bc_ (position 31). These 31 positions are chosen because they are more conserved and have more reliable alignment.
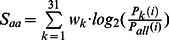
(3)where *P_k_*(*i*) is the probability of residue type *i* at the *k* ^th^ position of the WD40 repeat; *P_all_*(*i*) is the probability of a residue type *i* in all eukaryote proteins. To avoid zero probabilities, we use a pseudo-count of 0.0001 to all the frequencies. The weight *w*_k_ is the information entropy at *k* ^th^ position:



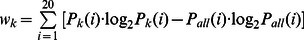
(4)
*S_loop_len_* is the sum of the scores for *l_da_, l_ab_* and *l_bc_*, which are the lengths of loops L_da_, L_ab_, L_bc_, respectively:
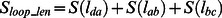
(5)


For each term in (5):
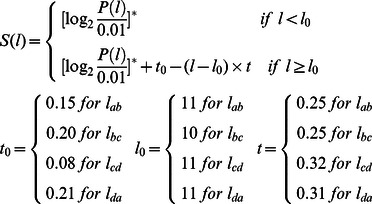
(6)


*P*(*l*) is the probability of loop length *l*. The raw *P*(*l*) is smoothed and the logarithmic curve is fitted to an analytical function **S1** (marked by superscript “*”). For the same reason, a pseudo-count of 0.01 was added to all the frequencies of the loop length. Because long loops in crystal structures are very rare, the accurate statistical estimation of the distribution of long loops is difficult to obtain. To penalize long loops that have almost no appearances in the crystal structures, an empirical linear penalty function was added to the loop score *S*(*l*). The term *t*_0_ is selected to smooth the transition between the fitted function and the linear function. The intercept *l*_0_ is the loop length when the score in the fitted curve is lower than 1. And the slope *t* is adjusted according to the feedback of the secondary structure prediction result. The detailed values of *S*(*l*) are listed in **Table S2 and TableS3**. The final curves of the fitted loop length scoring functions are shown in **Figure S2.**

*S_corr_* is added to the scoring function if there is pentad, tetrad or triad in a repeat: 
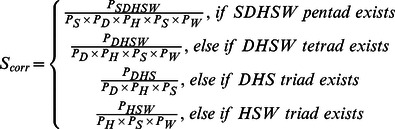
(7)


In equation (7), *P_SDHSW_* is the probability of Ser/Thr, Asp, His, Ser/Thr and Trp existing simultaneously at five certain positions, obtained from the training set. Here, the subscript *S* represents both amino acids Ser and Thr. The five positions for SDHSW pentads are Sc1, Lcd2, Lda3, Sb4 and Sc5, respectively.

For each position in the strands, *S_PSIPRED_* is calculated as shown in equation (8).
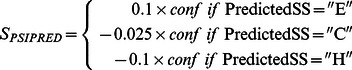
(8)


The result of PSIPRED is mainly used for the prediction of strand d in the repeat. In equation (8), the PSIPRED’s confidence value (*conf*) and predicted state (E: sheet, C: coil, H: helix) of each position are used in the predicted strands. The scaling parameters were manually adjusted to accept that *S_PSIPRED_* mainly affects strand d prediction. Because the sequence of strand d is much less conserved, the sequence logo is unable to identify strand d as efficient as other strands. Without *S_PSIPRED_*, WDSP can still predict over 90% of Sa, Sb and Sc correctly, but drops to 60% for Sd. In comparison, PSIPRED can predict all strands with similar accuracy. Thus, our purpose is to determine Sd with the use of *S_PSIPRED_*. The contribution of *S_PSIPRED_* is well balanced by applying the current coefficients (0.1, −0.025, −0.1) in equation 8. It contributes ∼65% for the score of Sd, but only 26.6% for the score of Sa, Sb and Sc and 16% of *S_repeat_*. As a result, it enhances the Q3 accuracy by 1.5% of WDSP in the prediction of secondary structures.

The scoring function for an entire WD40 domain contains the scores *S*_repeat_ of all individual repeats, together with the scores *S*(*l*_cd_) for the L_cd_ loops between these repeats:
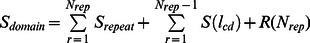
(9)


**Figure 5:**
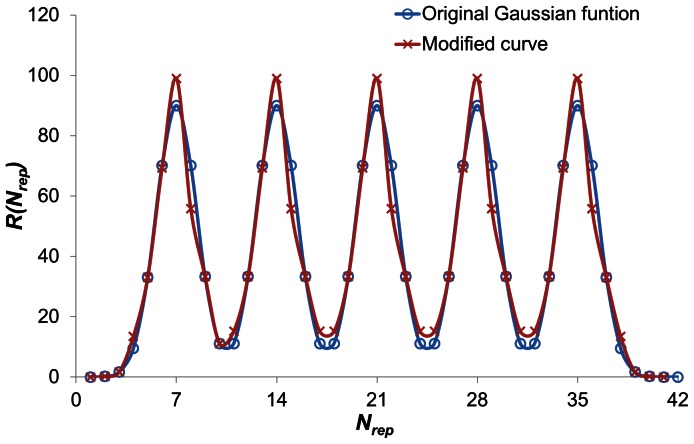
Curve of *R(N_rep_)*, which regulates the repeat number in the generated domain.

The additional term *R*(*N_rep_*) serves as a regulator for the repeat combination in the genetic algorithm to treat complete domains with regular 7-fold numbers of repeats. This term does not affect the score of generated repeats. A majority of WD40 domains are composed of 7 repeats, but 6 and 8 repeats are also possible. Because there are not enough available crystal structures for the reasonable statistics of WD40 domains with six and eight repeats, and no PDB structures for other repeat numbers, an accurate statistical analysis is not possible. For the consideration of multiple WD40 domains in one protein, we chose 5 overlapping Gaussian functions 
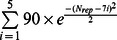
to give the original guess of the *R*(*N_rep_*) (blue curve in **Figure 5**). Then, we manually modified these values to get better repeat detection for the training set. This term is added to make the genetic algorithm engine more efficient in the repeat combination. And in the final step, *R(N_rep_)* was removed. As a result, only 3 out of 239 repeats are missed by WDSP by incorporating the modified curve (red curve in **Figure 5**, also see function **S2**).

### Flowchart of WDSP

**Figure 6:**
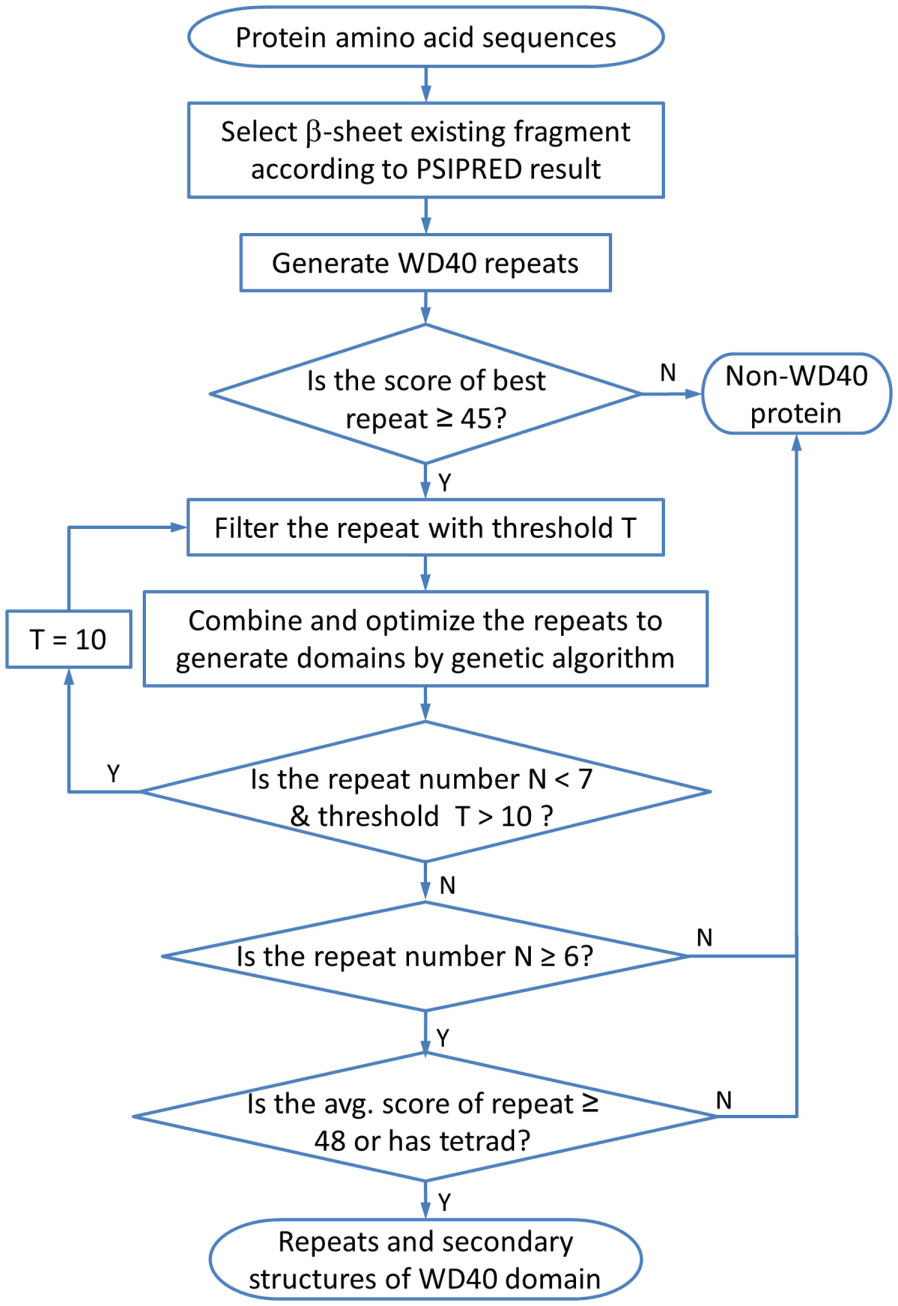
Flowchart of WDSP program.

**Figure 6** shows the flowchart of the WDSP program. The input of WDSP is the primary sequence. To increase the speed, the first step is to discard the sequence in the N- and C- terminus that are unlikely to fold into β-propeller based on PSIPRED predictions. This step is reliable because PSIPRED can provide reasonable secondary structure contents.

The second step is to generate all the possible WD40 repeats with *S*_repeat_>10. 10 is a fairly conservative value because the scores of true WD40 repeats in the PDB structures normally ranges from 30 to 150 (101 on average). Some repeats are discarded as they overlap with the other repeats with a score higher than 35. By these steps, there are normally 500–2000 repeats left in the library.

The third step is a preliminary exclusion of non-WD40 proteins. If the highest score of all generated repeats is less than 45, the sequence is defined not to be a WD40 protein. Otherwise, the combination of repeats will start.

To limit the size of the repeats pool for more efficient domain optimization, the generated repeats are further filtered by a threshold T:
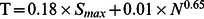
(10)where *S_max_* is the maximum score of all repeats in the library and *N* is the total number of repeats. The repeats are discarded as their scores are less than T. Usually, more than half of repeats are discarded in this procedure.

The genetic algorithm (GA) [49] was utilized to combine the remaining repeats into domains. In the GA process, each repeat is an individual in the first generation. Mutation, crossover and elongation operators are used to combine multiple repeats into one individual. Thus, the repeat number in individuals grows in the evolution process. If the best ten individuals in a generation converge to be identical one, the GA procedure converges. If the optimized domain has less than 7 repeats and the threshold T is larger than 10, the T value will be re-set to 10 and the GA process restarts. Finally, a sequence is determined to contain a WD40 domain(s) if the prediction fulfills two criteria: 1. it has more than 6 repeats; 2. the average score of repeats (*S_corr_* is not included) is not less than 48 or at least one DHSW tetrad is found.

**Figure 7:**
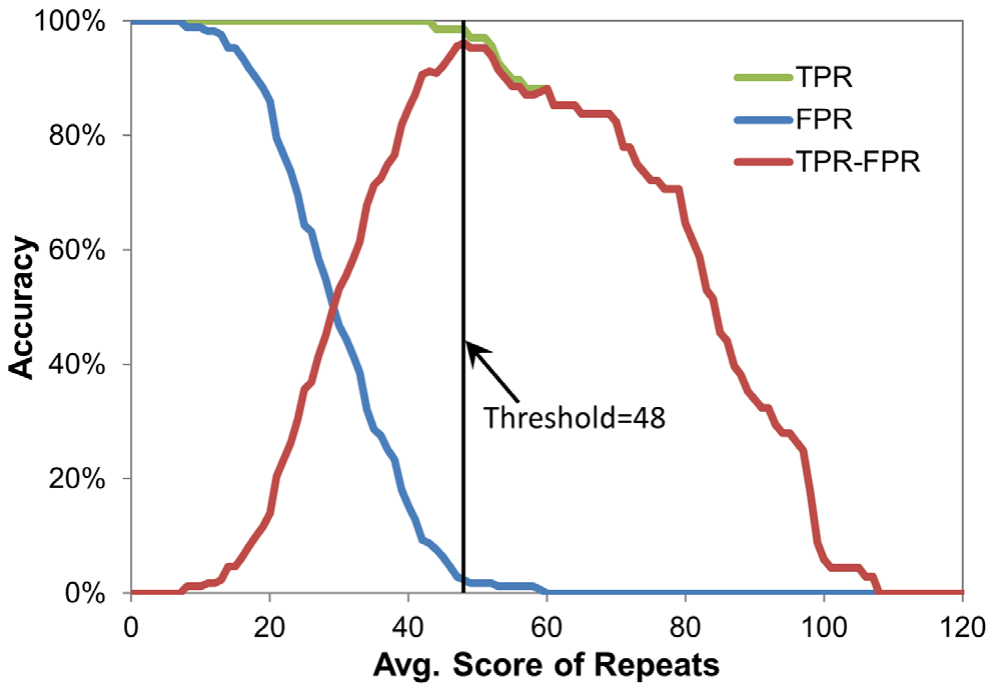
Percentage of true positive rate and false positive rate plotted versus the average score of repeats. TPR-FPR is the difference between the true positives and false positives, which reaches the highest value as the average score of repeats is above 48.

The threshold number 48 is determined based on the discrimination power between the true positive rate (TPR) and false positive rate (FPR). All proteins with crystal structures in the PDB database ranging from 250 to 2000 residues under a 95% sequence identity cutoff are used for the test. Totally, there are 13007 unique proteins in this group. The average value of *S_repeat_* in equation (1), without counting the *S_corr_*, is used to discriminate the True Positives (TP) and False Positives (FP). **Figure 7** shows the percentages of TP and FP versus different thresholds of the average score of repeats (ASr). As ASr is equal to 48, the optimal difference between the true positives and false positives (TPR-FPR) of 96.2% is achieved. This suggests that WDSP is able to distinguish between WD40 and non-WD40 proteins with a threshold value of 48.

## Results and Discussion

In this section, the advantages of sequence logo will be presented. Then, the validation of repeat detection and secondary structure prediction are further discussed. Finally, applications of WDSP are demonstrated.

### The New Sequence Logo

Recently, Stirnimann *et al.* [2] reported a similar sequence logo for WD40 repeats using the structural alignment of 12 WD40s as classified in SCOP [50]. Some well-known characters are found in both sequence logos, such as the GH dipeptide at the beginning or WD dipeptide at the end of S_c_. In addition, our new sequence logo can provide information for the DHSW tetrad, WD_b-a_ and WD_c-d_ β-bulges as well as some new structural features.

As shown in **Figure 4**, bulky residues at the 1^st^ (V = 49.2%, I = 29.0%, L = 6.3%) and the 4^th^ (V = 35.6%, L = 23.8%, I = 14.2%) positions of S_a_ encircle R_1_ and R_2_ in the WD_b-a_ β-bulges, respectively. As compared with the earlier sequence logo, they are more conserved and thus the corresponding letters are larger. At the meanwhile, Phe, Trp and Tyr are dominant at the end of S_a_ (F = 30.1%, W = 27.6%, Y = 8.4%). The S_a_ in Stirnimann’s assignment is shifted to the C-terminus by one residue.

In S_b_, the last three residues favor those with small side-chains (Ser, Gly, Ala, Thr). At the 4^th^ position, Ser and Thr are involved in DHSW tetrad. At the 5^th^ position, the dominant residues Gly, Ala, Ser and Cys are at the X position of WD_b-a_ β-bulge [36]. The reside at the end of S_b_ favors Ser, Gly, Thr and Asp because they play roles in initiating the compact β-turn connecting S_b_ and S_c_ [51], [52].

The 2^nd^ and 4^th^ positions in S_c_ more favor bulky residues (2^nd^: I = 33.9%, V = 29.3%, L = 18.4%; 4^th^: V = 29.7%, I = 26.8%, L = 23.8%). The 2^nd^ position residue often takes part in the hydrophobic core formation (**Figure 1C**) and the 4^th^ position residue is at the X position of WD_c-d_ β-bulge, where bulky residues are normally favorable [37], [38].

S_d_ is less conserved. The residue at the 1^st^ position favors charged side-chains, such as Lys and Glu, and polar side-chains, such as Thr, Ser, Gln and Asn. At the 3^rd^ and 4^th^ positions are the R_1_ and R_2_ residues of the WD_c–d_ β-bulge. R_1_ and the last residue of S_d_ favor bulky residues.Pro also has a significant preference at the 2^nd^ position(39.6%) of L_ab_ and at the 6^th^ position(13.0%) of L_da_ (see **Table S4**).

### Validation of WD40 Repeats Detection

In order to test the capability of WDSP in identifying WD40 repeats, a jack-knife test has been carried out to predict repeats composed of 33 WD40s in our training set. The results are further compared with currently well-accepted methods; UniProt, SMART, Pfam and PROSITE. Both loose and tight criteria are applied for the evaluation. In the loose criterion, a WD40 repeat is considered successfully identified if S_a_, S_b_ and S_c_ are found in the sequence. In the tight criterion, a predicted WD40 repeat is required to contain S_a_, S_b_, S_c_ and S_d_.

**Figure 8:**
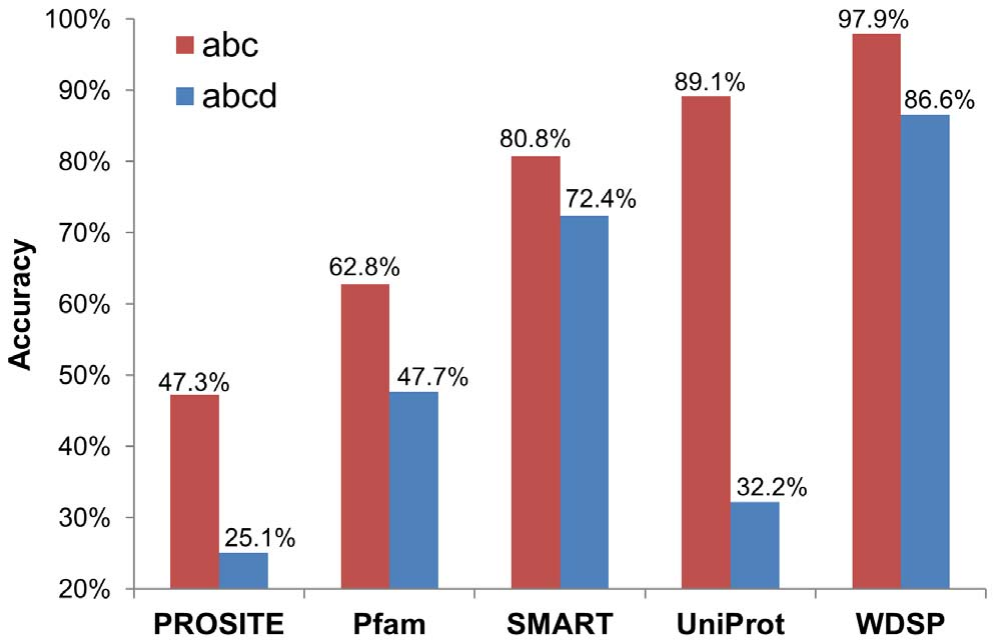
Accuracy of WD40 repeats detection by PROSITE, Pfam, SMART, UniProt and the jack-knife results of WDSP with the use of the loose and tight criteria. The red bar represents the loose criterion: only containing S_a_, S_b_ and S_c_; while the blue bar represents the tight criterion: including all four strands.

As shown in **Figure 8**, WDSP has higher accuracy than UniProt and SMART, although their performances are much better than those PROSITE and Pfam. Under the loose criterion, WDSP is able to identify 234 out of 239 repeats (97.9% accuracy). In comparison, UniProt and SMART have 89.1% and 80.8% accuracy, respectively. For the tight criterion, the advantage of WDSP is further demonstrated. WDSP correctly identified 207 out of 239 repeats (86.6%). In comparison, SMART and UniProt only have 72.4% and 32.2% accuracy, respectively. The remarkable reduction indicates that these methods have defects on determining the strand d in WD40 repeat. Although UniProt can identify most of WD40 repeats in the loose criterion, a majority of them are shorter than their lengths in reality. The detailed results are shown in **Table S5**. As a result, WDSP has a better performance in WD40 repeat identification, especially by the tight criterion.

### Validation of Secondary Structure Prediction

**Figure 9:**
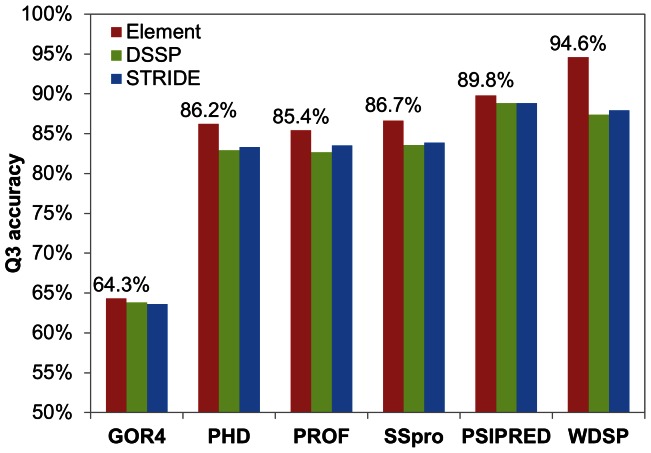
GOR4, PHD, PROF, SSpro, PSIPRED and WDSP are compared to predict the secondary structures of the 33 WD40s. The secondary structure assignment by the structural element, DSSP and Stride are used as references.

The accuracy of secondary structure prediction was compared with five well-known secondary structure prediction methods, GOR4, PHD, PROF, SSpro and PSIPRED. Q3 criterion [29] was used to evaluate the secondary structure prediction. **Figure 9** shows the Q3 values achieved by the different methods. For a comprehensive comparison, DSSP, STRIDE and structural-feature-based secondary structure assignments were applied to evaluate the predictions. As expected, WDSP has the highest accuracy (Q3 = 94.6%) using the structural-feature-based assignment. As the secondary structure is assigned by DSSP or STRIDE, only PSIPRED performs slightly better than WDSP. Thus, WDSP is excellent in predicting the secondary structure for the WD40 domain. Interestingly, although all these methods except for WDSP were trained based on DSSP or STRIDE, they all got better performance under the structural feature-based secondary assignment. It suggests that the structural-feature-based assignment may provide more representative secondary structures of WD40s than DSSP and STRIDE.

**Figure 9** also shows most of methods have good performance for the secondary structure prediction for the WD40 domain. PSIPRED and WDSP have almost reached the general upper limit of the prediction accuracy of 88% [53]. This is because WD40 domains only contain two types of secondary structures, β-strands and loops. The upper limit should be higher than the normal value. Another possibility is that all the above methods have utilized their crystal structures in the training set. Therefore, the accuracy would be the reproduction rate.

The ASr can be further used to estimate the secondary structure accuracy. A good correlation (R^2^ = 0.64) has been found between the ASr and their Q3 accuracy of 33 WD40 proteins in the training set (**Figure S3**). It suggests that we may roughly estimate the Q3 accuracy by the ASr score.

### The Jack-knife Test of Secondary Structure Prediction

**Figure 10:**
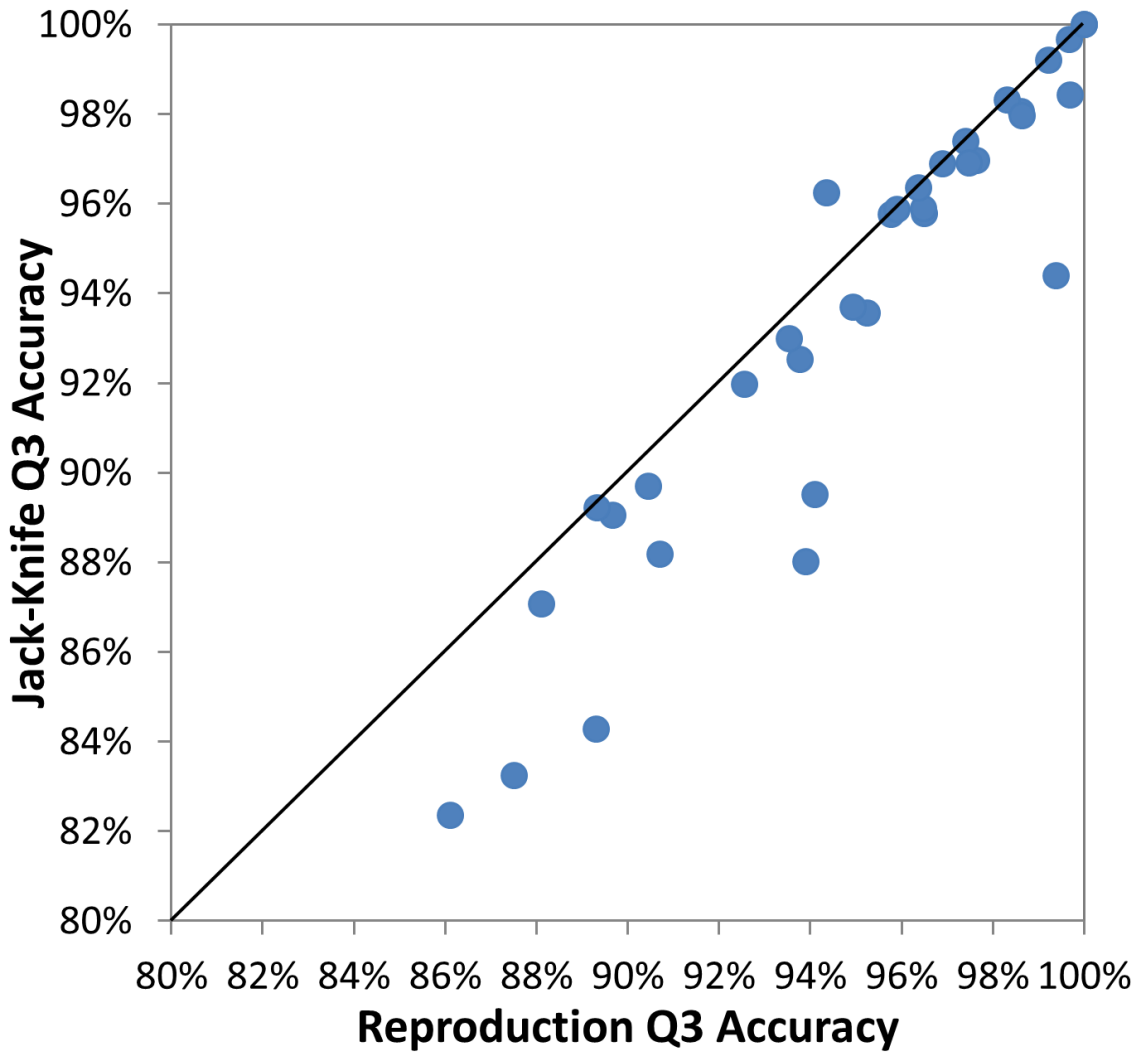
Jack-knife results versus the reproduction results in the 33 PDBs.

To exclude the overestimation of accuracy by reproduction, a jack-knife test has been carried out. The test utilizes 32 WD40s as the training set and the left out one WD40 as the test set. This procedure has been repeated 33 times until every protein is predicted once. The resultant secondary structures are compared with the reproductive prediction. As shown in **Figure 10**, the x and y-axis show the reproductive rate and the jack-knife result, respectively. Although a part of reproductive accuracies seem to be higher, a majority of circles are very close to the diagonal. The average Q3 of jack-knife test is about 93.7%. The accuracy is almost identical to the average reproduction accuracy of 94.6% in **Figure 9**. Thus, the accuracy of secondary structure prediction is apparently not overestimated.

The good performance in the jack-knife tests can be rationalized by two reasons. 1. Although the selected proteins in our dataset are highly diversified in sequence, the residue frequencies are relatively stable on the basis of structural-feature-based alignment. Thus, the scoring function would be invariant. 2. The structural features, such as tetrads, β-bulges and total number of blades, are fairly conserved across the family. Thus, their preferences can be derived from limited protein structures. Both are the major components of *S_repeat_* in scoring function (1).

### The Prediction of WD40 Domains in the Swiss-Prot Database by WDSP

Besides the accurate prediction of WD40 repeats and secondary structures, one of our goals is to identify the missing/new WD40 repeats in the known/unknown WD40 proteins.

Before we tested its capability in identifying WD40 proteins from the UniProt protein database, an estimation of prediction accuracy was carried out. Two datasets were prepared for tests. As shown in **Table 1**, the positive dataset contains 1402 proteins, which are composed of WD40 domains with a sequence identity cutoff of 50%. Only 16 WD40 proteins are not identified by WDSP, the False Positive rate is around 1.14%. In the negative dataset, it’s composed of 2496 all-β proteins and 4669 all-α, α+β, α/β proteins. The homologues are deleted with the sequence identities of more than 30%. Only 4 proteins with all-β sheets are predicted to be WD40s. None of proteins with α-helix are falsely predicted to be WD40s. Thus, the false positive and the false negative are controlled at a very low level.

**Table 1:** Evaluation of WDSP in predicting unknown proteins.

	WD40^a^	Non-WD40^b^		
		all-β	all-α, α+β, α/β	
Proteins in Dataset	1402	2496	4669	
WDSP mistakes	16	4	0	

a The positive WD40 protein dataset has 1402 relatively confident WD40 proteins that were selected by using the query in UniProt database: family: “wd repeat” AND domain: “wd repeats” AND annotation:(type:repeat wd) AND database:(type:smart wd) AND database:(type:pfam wd).

b The negative dataset consist of 2496 non-WD40 all-β proteins from the SCOP database and 4669 non-WD40 all-α proteins, α+β proteins or α/β proteins. As for all-β proteins, the homologues are deleted if the sequence identities are larger than 50%. The homologues of all-α proteins, α+β proteins and α/β proteins are removed with the identity cutoff at 30%.

WDSP was further utilized to detect WD40 proteins in 271,654 non-redundant proteins with sequence length range from 250–2000 residues selected from the Swiss-Prot database (release 2012_07). **Table 2** summarizes the performances by PROSITE, Pfam, SMART, UniProt and WDSP in the WD40 repeat detection. Several remarkable advantages of WDSP are able to be observed.

**Table 2:** Comparison of five methods in detecting WD40 repeats/domains/proteins from Swiss-Prot database with sequence length less than 2000 residues.

	PDB	WDSP	PROSITE	Pfam	SMART	UniProt
Repeats	239	17344	6287	8057	12440	14517
Domains^a^	34	2600/2600^b^	1827/341 b	1977/599 b	2255/1428 b	2473/1809 b
Proteins^a^	33	2277/2277 b	1813/327 b	1952/574 b	2135/1308 b	2250/1586 b
Avg repeats/domain	7.0	6.7	3.4	4.1	5.5	5.9

a Except WDSP, if one WD40 repeat is detected, the protein is classified as containing at least one WD40 domain. For WDSP, each WD40 domain and WD40 protein has at least 6 WD40 repeats. If the total number of repeats in a protein has exceeded 8, 16, 24 and 32, two, three four and five WD40 domains are detected for the method.

b Number of domains/proteins with at least 6 WD40 repeats, which use the same rules as WDSP in determining WD domains/proteins.

Firstly, WDSP can identify many more WD40 repeats than the other methods. 17344 WD40 repeats are identified in total. Among the four other methods, UniProt has the best performance. However, only 14517 WD40 repeats are annotated, which is about 20% less than WDSP.

Secondly, WDSP identified 2600 WD40 domains with at least 6 repeats. There are 2473 WD40 domains included in UniProt, which is slightly fewer than predicted by WDSP. More significantly, only 1809 have more than 5 repeats in UniProt. On average, each WD40 domain is estimated to have 6.7 repeats by WDSP. The value 6.7 is close to the observed average repeat number 7.0 in WD40 protein crystal structures. But each WD40 domain is estimated to have 5.9 and 5.5 repeats by UniProt and SMART, respectively. As shown in **Table 2**, PROSITE and Pfam have a much lower performance in identifying both WD40 domains and repeats. Therefore, WDSP can identify more WD40 domains with more WD40 repeats.

**Figure 11:**
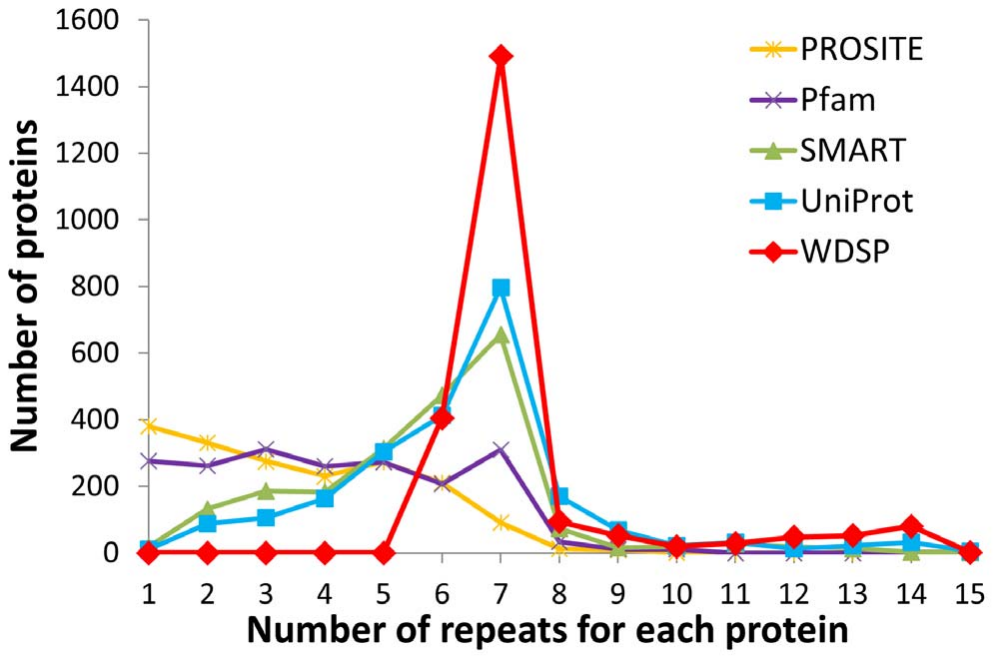
Repeat number distributions of WD40 proteins identified by PROSITE, Pfam, SMART, UniProt and WDSP from 271,654 proteins.

It’s well-known that WD40 domains are composed of six to eight, but mostly seven repeats [54]. Thus, we further compared WD40 repeat distributions by the five methods. **Figure 11** shows 18%, 65%, and 4% WD40 domains are predicted to have six, seven, and eight repeats by WDSP, respectively. PROSITE and Pfam have even distributions from one to five repeats. Only a few proteins are predicted to have seven repeats by PROSITE. By Pfam, even repeated proteins are found slightly more often than those with one to six repeats. Apparently, the number of WD40 repeats is considerably underestimated in a WD40 domain by these methods. As a matter of fact, SMART and UniProt have a better performance than PROSITE and Pfam in identifying WD40 repeats. However, 30% and 39% WD40 proteins are predicted to have less than six repeats by SMART and UniProt, respectively.

More significantly, WDSP predicts a large number of proteins with multiple WD40 domains. According to prediction, 280 proteins have two WD40 domains, 12 proteins have three WD40 domains and three proteins have four WD40 domains. The repeats in these multiple-domain proteins are underestimated by the other four methods as well.

**Figure 12:**
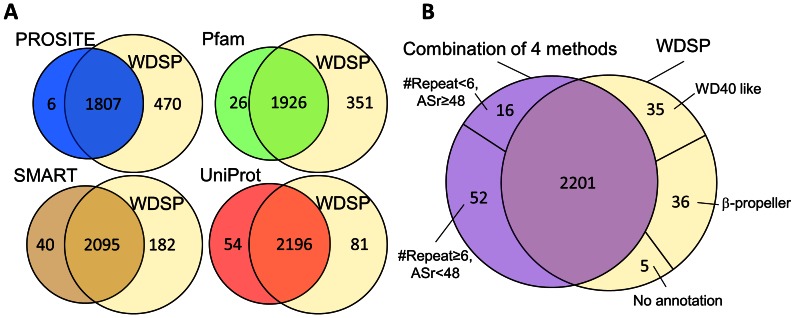
The comparisons of predictions by WDSP. **(A) Comparison of WDSP with four methods in WD40s detection.** (B) Comparison of WDSP and the combination of PROSITE, Pfam, SMART and UniProt in WD40s detection.

**Figure 12A** further demonstrates the similarities and differences between WDSP and the other four methods in WD40 domain/protein detection. 1807 WD40 proteins are commonly identified by both WDSP and PROSITE. 470 WD40 proteins are identified by WDSP and 6 proteins are missed in the comparison. Although the majority of WD40 proteins are commonly identified, 351, 182 and 81 WD40 domains failed in being identified by Pfam, SMART and UniProt, respectively. As compared with Pfam, SMART and UniProt, WDSP fails in identifying 26, 40 and 54 WD40 domains, respectively. Thus, WDSP has a better performance in identifying WD40 domains.

Comparing with the combination of the four methods, only 76 potential WD40 proteins are solely detected by WDSP (**Figure 12B**). Among these proteins, 35 of them are classified as WD40-like proteins in InterPro [55], SUPERFAMILY [56] or Gene3D [57] database. Another 36 proteins are predicted to be other β-propeller proteins as well. However, the tight definition is unavailable to differentiate WD40 proteins and the normal β-propellers. The remaining five proteins have no annotation available and might be new WD40 proteins (**Table S6**).

**Figure 13:**
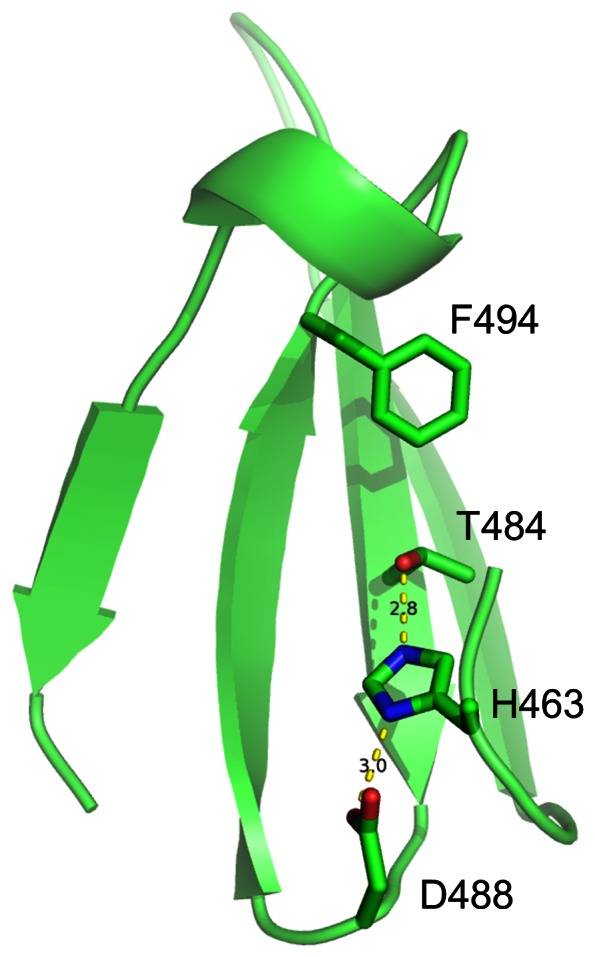
Hydrogen-bonded triad formed by D488-H463-T484 in tau91. In typical WD40s, F494 is always replaced by W or Y. Such triad is a special structural feature for WD40 protein family.

68 proteins are cannot to be identified by WDSP (**Figure 13B**). By the analysis, 16 of them are identified to have less than six repeats, which are considered incomplete WD40 domains. This may be due to the incomplete sequences. The remaining 52 proteins are predicted with ASr less than 48 (**Table S7**). By the criteria, they are not classified to be WD40 proteins by WDSP.

A specific protein in the PDB database, tau91 from *S. cerevisiae* with a 7-bladed β-propeller structure (PDB_ID:2J04) [58], was detected to be a WD40 protein by WDSP. None of four methods has classified it to be a WD40 protein. Undoubtedly, tau91 is quite different from the typical WD40 proteins in the sequence. But it shares some WD40 conserved structure features in common. Although Tau91 is short of a DHSW tetrad, a hydrogen-bounded triad is formed by D488-H463-T484 as shown in **Figure 13**. In the typical WD40 proteins, F494 is usually replaced by W or Y to form the D488-H463-T484-W/Y494 tetrad. According to the previous results, D488-H463-T484 is able to provide remarkable stability [34], [35]. Meanwhile, X and R_2_ of WD_b-a_ β-bulges have the similar residue preferences. Some R_1_ residues, such as W367 and L468, are protruded to the surface and readily for protein-protein interaction. By incorporating non-local information of structural features, WDSP overcomes the sequence diversity and classifies tau91 as a WD40 domain (**Figure S4**).

Since we have tested the general performance of WDSP in predicting Swiss-Prot proteins, we used a WD40 protein LRRK2 as an example to show how WDSP performs in predicting difficult targets. LRRK2 is a multi-domain protein whose mutations are frequently found in familial and sporadic Parkinson’s disease [39], [42]. Thus, LRRK2 could be a potential therapeutic target for drug design [43]. Currently, the crystal structure of LRRK2 WD40 domain is not available. Although LRRK2 is known for years to contain a WD40 domain, until now no existing method has given a high quality prediction of the WD40 repeats and its detailed topology.

**Figure 14:**
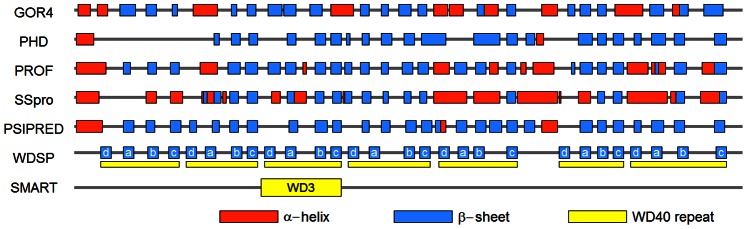
Secondary structure prediction and repeat detection for LRRK2 protein by various methods. Red, blue and yellow bars indicate the predicted α-helix, β-strand and WD40 repeat. For WDSP, each predicted β-strand is annotated with strand IDs. Among the competing repeat detection methods, only SMART gives one positive result.

**Figure 15:**
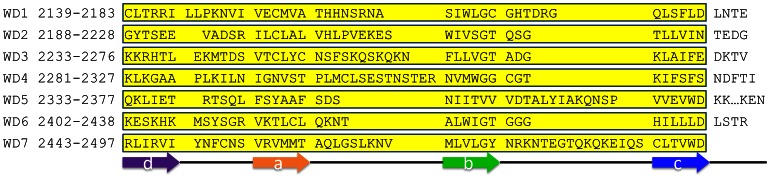
WDSP predicts the secondary structure of LRRK2 protein. The strand IDs are depicted below. Residues in the yellow boxes are detected repeats. The starting and ending positions for each repeat are shown in the left.

**Figure 14** shows the secondary structure prediction and repeat detection results of LRRK2 protein by different existing methods. The different methods give very variable predictions. Some positions predicted to be a β-strand by one method are predicted to be a α-helix by another method. Almost all predicted β-strands by WDSP are supported by one or more other methods, which indicate that its prediction is likely to be reliable. Among the existing WD40 repeat detection methods, PROSITE, Pfam, SMART, REP and UniProt, only SMART has identified one WD40 repeat (the 3^rd^ repeat). However, WDSP identified all 7 repeats in LRRK2. **Figure 15** depicts the detailed description of secondary structure and the topology of the WD40 domain. The prediction can provide some useful information for experimentalists and can also lead to accurate 3D structure prediction directly.

### Conclusions

Protein structure is the basis to understand the interaction of WD40-repeat proteins. Accurate secondary structure prediction is a bridge to 3D structure prediction. A number of methods are available to predict either WD40 repeats or secondary structures, which provide the preliminary information for biological studies and structure prediction. The currently available methods usually have defects in identifying comprehensive repeats/sequences for the WD40 domains because these domains have extremely diversified sequences due to their variable binding capabilities. In addition, the predictions are unable to provide biofunctional/structural information directly. Here, a method, WDSP, has been developed to identify WD40 repeats and predict its secondary structure simultaneously. By incorporating the specific structure/function-sequence information, WDSP is able to overcome the problem of diversified sequences, variable loop lengths and even identify atypical WD40 domains. Thus, WDSP provides a useful tool for structure/function prediction of WD40 domains. The method also provides a novel solution for specific protein families, especially for that composed of repeated motifs. As long as the structure-sequence correlation can be correctly recognized, the repeats and secondary structure can be predicted accurately.

## Supporting Information

Figure S1 **Correlation and similarity of amino acid frequency at every position in 10 independent tests.**
(DOCX)

Figure S2 **Fitted curves for the score of loop length.**
(DOCX)

Figure S3 **Q3 accuracy versus the threshold of average score of repeats.**
(DOCX)

Figure S4 **Predicted secondary structure of tau91 protein (PDB code: 2J04 chain D).**
(DOCX)

Table S1 **Pairwise sequence identities of selected 33 WD40 proteins (34domains, use PDB codes as names).**
(DOCX)

Table S2 **Parameters used in function S1.**
(DOCX)

Table S3 **The final scores of loop length in the score function.**
(DOCX)

Table S4 **Residue frequencies of every position in the sequence logo.**
(DOCX)

Table S5 **Repeat detection comparison by different methods.**
(DOCX)

Table S6 **76 potential WD40 proteins are only detected by WDSP.**
(DOCX)

Table S7 **68 proteins are unable to be identified by WDSP.**
(DOCX)
