# Supplementary material for: A Method for WD40 Repeat Detection and Secondary Structure Prediction
Source: PLoS One. 2013 Jun 11;8(6):e65705. doi: 10.1371/journal.pone.0065705 (PMC3679165; doi:10.1371/journal.pone.0065705)
Supplement: Figure S1 — Correlation and similarity of amino acid frequency at every position in 10 independent tests. (DOCX) [file pone.0065705.s001.docx]

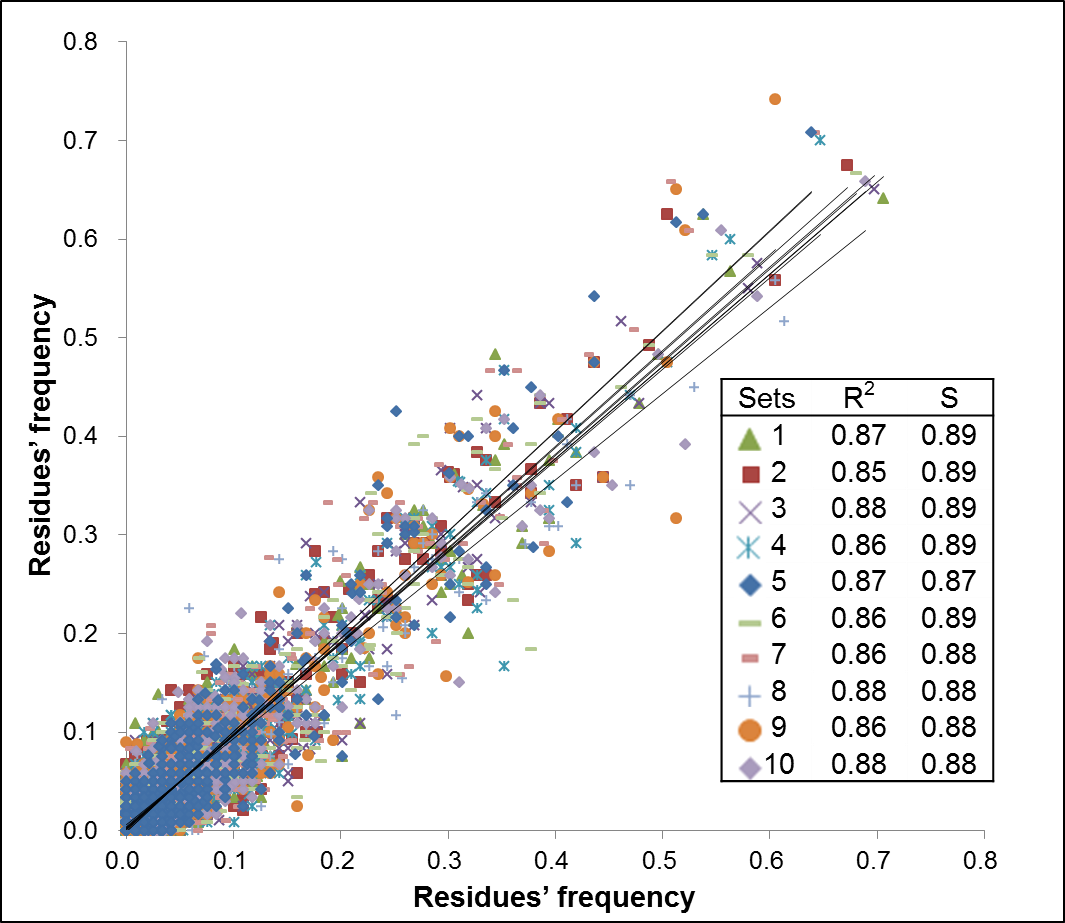


**Figure S1**. Correlation and similarity of amino acid frequency at every position in 10 independent tests. The embedded table describes R^2^ and S values in every test.
