# Supplementary material for: A Method for WD40 Repeat Detection and Secondary Structure Prediction
Source: PLoS One. 2013 Jun 11;8(6):e65705. doi: 10.1371/journal.pone.0065705 (PMC3679165; doi:10.1371/journal.pone.0065705)
Supplement: Figure S2 — Fitted curves for the score of loop length. (DOCX) [file pone.0065705.s002.docx]

1. Overlapped the fitted curves on the original one**.**


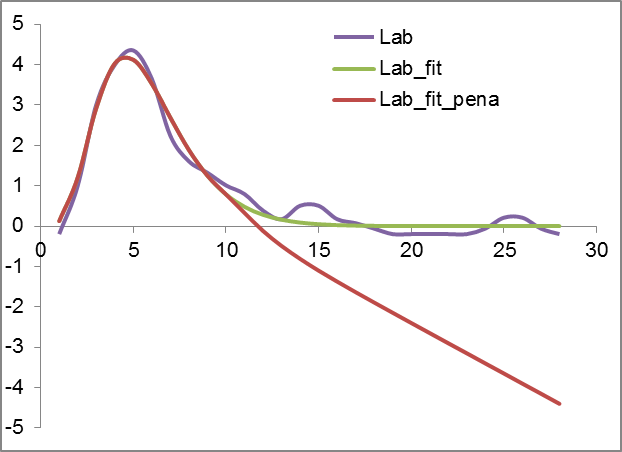

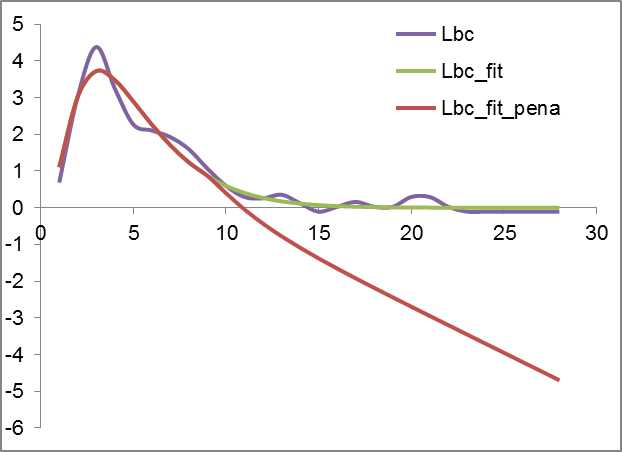


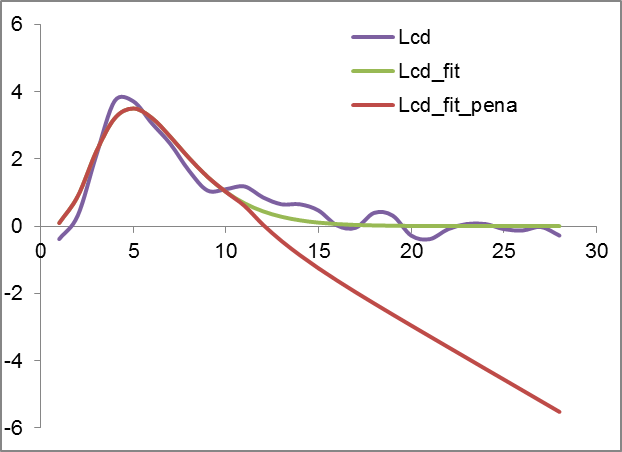

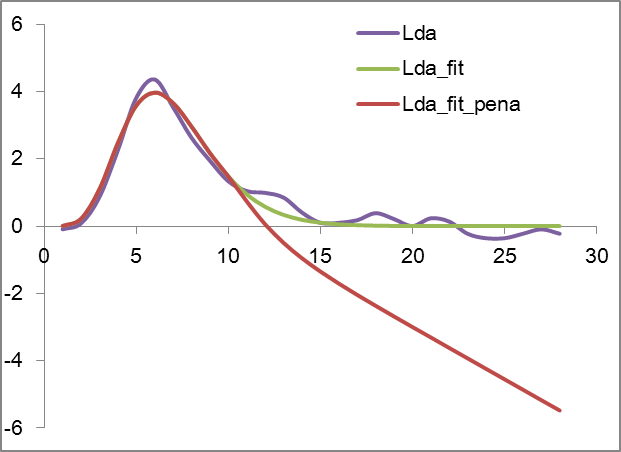


**Figure S2**. Fitted curves for the score of loop length. L_ab_, L_bc_, L_cd_ and L_da_ (L_xx_) represent the original logistic curves for loop length. L_xx__fit represent the fitting curve in the function (**S1**). L_xx__fit_pena represent the final scoring function after adding the linear penalty.

$\begin{matrix} R(N_{rep})=(\text{ 0, 0, 2, 14, 33, 69, 99, } \\ \text{ 56, 33, 15, 15, 38, 69, 99, } \\ \text{ 56, 33, 15, 15, 38, 69, 99,} \\ \text{ 56, 33, 15, 15, 38, 69, 99, } \\ \text{ 56, 33, 15, 15, 38, 69, 99,} \\ \text{ 56, 33, 14, 2, 0, 0, 0})\text{ } \\ \text{ }for\text{ }N_{rep}\text{ }from\text{ }1\text{ }to\text{ }42 \end{matrix}$ (**S2**)
