# Supplementary material for: A Method for WD40 Repeat Detection and Secondary Structure Prediction
Source: PLoS One. 2013 Jun 11;8(6):e65705. doi: 10.1371/journal.pone.0065705 (PMC3679165; doi:10.1371/journal.pone.0065705)
Supplement: Figure S3 — Q3 accuracy versus the threshold of average score of repeats. (DOCX) [file pone.0065705.s003.docx]

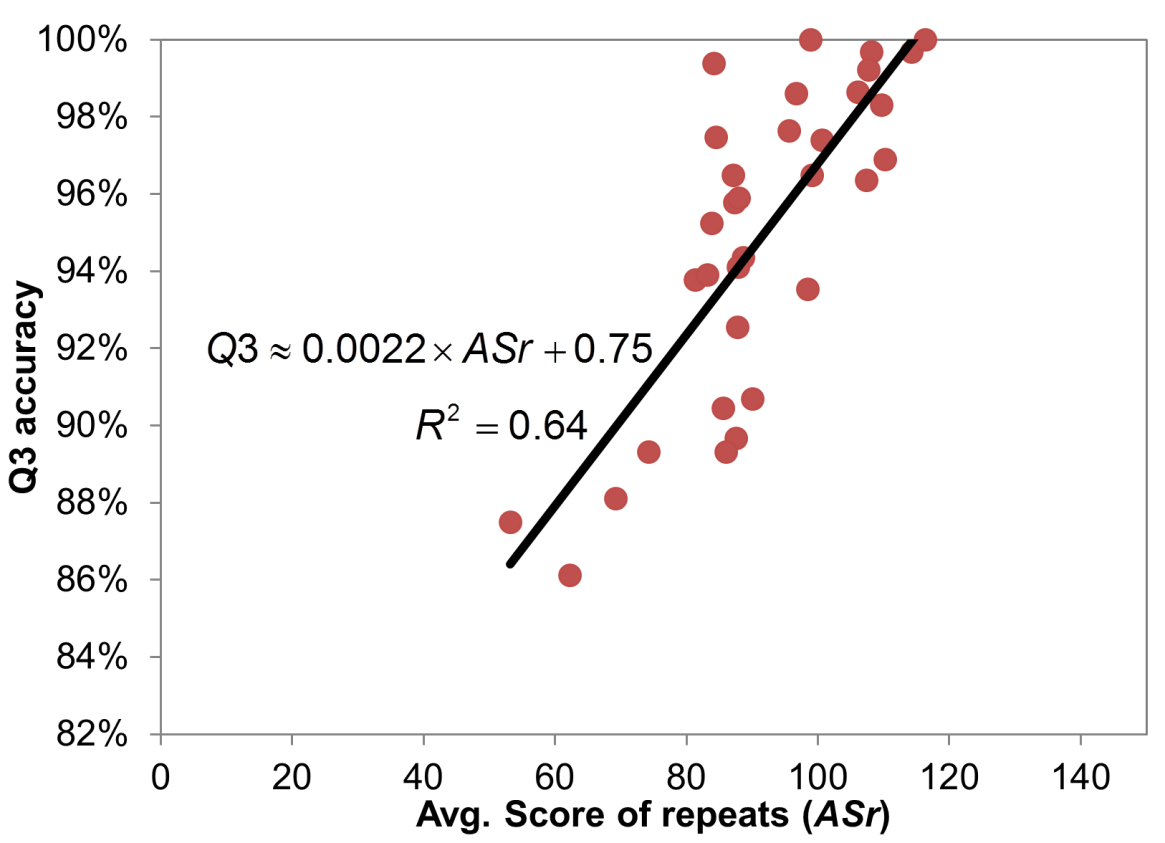


**Figure S3**. Q3 accuracy versus the threshold of average score of repeats. A linear correlation (R^2^=0.64) of between the average score of repeats(ASr) and Q3 accuracy is observed. Thus we are able to rough estimate the Q3 accuracy using the ASr.
