# Supplementary material for: A Method for WD40 Repeat Detection and Secondary Structure Prediction
Source: PLoS One. 2013 Jun 11;8(6):e65705. doi: 10.1371/journal.pone.0065705 (PMC3679165; doi:10.1371/journal.pone.0065705)
Supplement: Figure S4 — Predicted secondary structure of tau91 protein (PDB code: 2J04 chain D). (DOCX) [file pone.0065705.s004.docx]

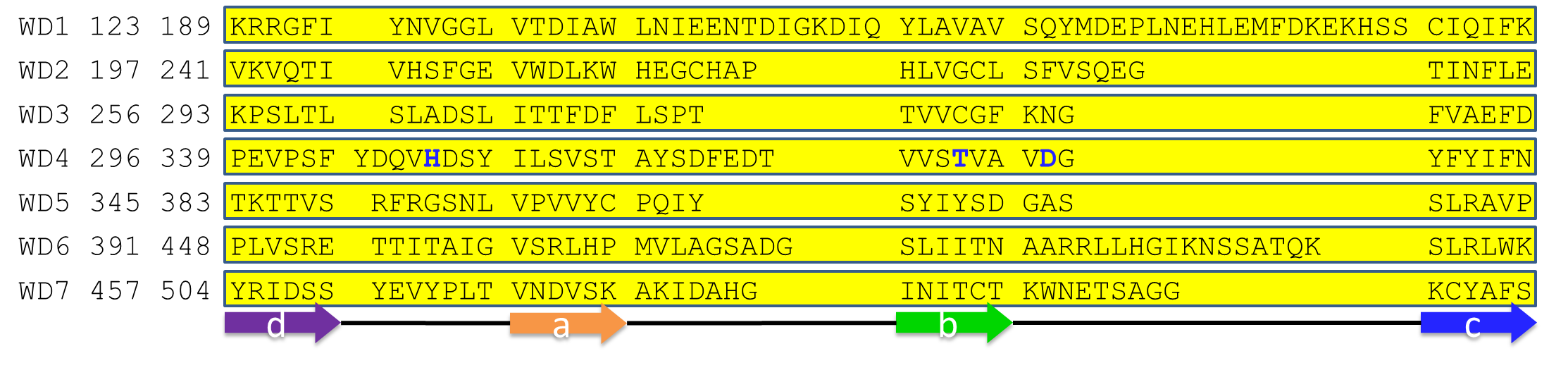


**Figure S4**. Predicted secondary structure of tau91 protein (PDB code: 2J04 chain D). None of WD40 repeat is identified by one of UniProt, SMART, Pfam, REP, PROSITE). By WDSP, most of the strands are correctly predicted except a shift in the first S_a_. Residues involved in triad H-bond network are highlighted in blue color, which are consistent with its crystal structure.
