# Supplementary material for: A Method for WD40 Repeat Detection and Secondary Structure Prediction
Source: PLoS One. 2013 Jun 11;8(6):e65705. doi: 10.1371/journal.pone.0065705 (PMC3679165; doi:10.1371/journal.pone.0065705)
Supplement: Table S1 — Pairwise sequence identities of selected 33 WD40 proteins (34domains, use PDB codes as names). (DOCX) [file pone.0065705.s005.docx]

**Table S1**. Pairwise sequence identities of selected 33 WD40 proteins (34domains, use PDB codes as names). Only the WD40 domain of each protein is used in the Needleman-Wunsch global alignment.

| % | 1P22 | 3EWE | 2HES | 2AQ5 | 1SQ9 | 3ZWL | 3GFC | 1K8K | 2PM9 | 1NEX | 1R5M | 2VDU | 3ACP | 3BG1 | 3EI3 | 3ODT | 2OVP | 3EMH | 3I2N | 3MMY | 1NR01 | 1NR02 | 1VYH | 1YFQ | 3OW8 | 4A11 | 4AEZ | 3MKQ | 3FRX | 1XHM | 3IIW | 1GXR | 3DW8 | 1ERJ |
| --- | --- | --- | --- | --- | --- | --- | --- | --- | --- | --- | --- | --- | --- | --- | --- | --- | --- | --- | --- | --- | --- | --- | --- | --- | --- | --- | --- | --- | --- | --- | --- | --- | --- | --- |
| 1P22 | 100 | 17 | 18 | 18 | 15 | 16 | 18 | 16 | 17 | 27 | 20 | 18 | 19 | 15 | 16 | 21 | 28 | 22 | 15 | 15 | 20 | 18 | 23 | 18 | 18 | 19 | 18 | 20 | 20 | 18 | 18 | 19 | 17 | 23 |
| 3EWE | 17 | 100 | 19 | 16 | 19 | 16 | 19 | 16 | 19 | 13 | 15 | 16 | 13 | 30 | 16 | 17 | 17 | 19 | 18 | 16 | 14 | 19 | 19 | 17 | 22 | 14 | 18 | 21 | 18 | 19 | 15 | 18 | 13 | 16 |
| 2HES | 18 | 19 | 100 | 17 | 16 | 18 | 19 | 18 | 16 | 17 | 18 | 18 | 17 | 23 | 17 | 18 | 19 | 22 | 17 | 15 | 17 | 18 | 21 | 18 | 22 | 20 | 18 | 21 | 17 | 19 | 18 | 16 | 15 | 21 |
| 2AQ5 | 19 | 16 | 17 | 100 | 16 | 18 | 17 | 19 | 17 | 16 | 16 | 18 | 18 | 19 | 15 | 18 | 20 | 19 | 17 | 19 | 14 | 16 | 21 | 18 | 15 | 18 | 16 | 18 | 18 | 20 | 18 | 20 | 16 | 19 |
| 1SQ9 | 15 | 20 | 16 | 16 | 100 | 18 | 16 | 16 | 17 | 17 | 16 | 15 | 18 | 16 | 18 | 18 | 17 | 19 | 20 | 21 | 20 | 16 | 20 | 18 | 23 | 17 | 18 | 15 | 17 | 16 | 15 | 19 | 17 | 20 |
| 3ZWL | 16 | 16 | 18 | 18 | 18 | 100 | 14 | 16 | 16 | 16 | 16 | 17 | 16 | 17 | 18 | 17 | 16 | 20 | 17 | 16 | 17 | 19 | 17 | 16 | 19 | 17 | 16 | 19 | 20 | 17 | 17 | 17 | 14 | 18 |
| 3GFC | 18 | 19 | 19 | 17 | 16 | 15 | 100 | 19 | 17 | 18 | 20 | 19 | 18 | 15 | 19 | 19 | 15 | 19 | 17 | 19 | 17 | 20 | 23 | 18 | 18 | 21 | 18 | 16 | 17 | 23 | 18 | 18 | 17 | 20 |
| 1K8K | 16 | 16 | 18 | 19 | 16 | 16 | 19 | 100 | 18 | 15 | 18 | 19 | 16 | 20 | 18 | 17 | 18 | 17 | 17 | 18 | 16 | 17 | 16 | 18 | 18 | 17 | 18 | 15 | 15 | 19 | 17 | 19 | 17 | 19 |
| 2PM9 | 17 | 19 | 16 | 17 | 17 | 16 | 17 | 18 | 100 | 17 | 20 | 16 | 16 | 17 | 17 | 18 | 17 | 19 | 16 | 16 | 19 | 15 | 19 | 17 | 19 | 20 | 22 | 17 | 17 | 16 | 18 | 18 | 18 | 19 |
| 1NEX | 27 | 13 | 17 | 16 | 17 | 16 | 18 | 15 | 17 | 100 | 20 | 16 | 19 | 17 | 18 | 20 | 31 | 19 | 15 | 17 | 17 | 15 | 20 | 19 | 18 | 16 | 16 | 16 | 19 | 19 | 19 | 15 | 16 | 22 |
| 1R5M | 20 | 15 | 18 | 16 | 16 | 16 | 20 | 18 | 20 | 20 | 100 | 16 | 16 | 18 | 20 | 14 | 19 | 18 | 17 | 15 | 18 | 15 | 17 | 17 | 20 | 17 | 18 | 17 | 19 | 19 | 17 | 18 | 15 | 16 |
| 2VDU | 18 | 16 | 18 | 18 | 15 | 17 | 19 | 19 | 17 | 16 | 16 | 100 | 19 | 17 | 16 | 19 | 18 | 17 | 16 | 16 | 19 | 17 | 16 | 17 | 16 | 16 | 17 | 17 | 17 | 16 | 18 | 17 | 16 | 15 |
| 3ACP | 19 | 13 | 17 | 18 | 18 | 16 | 18 | 16 | 16 | 19 | 16 | 19 | 100 | 14 | 20 | 20 | 18 | 17 | 17 | 17 | 17 | 16 | 17 | 15 | 19 | 18 | 15 | 17 | 16 | 17 | 17 | 16 | 18 | 19 |
| 3BG1 | 15 | 30 | 23 | 19 | 16 | 17 | 15 | 20 | 17 | 17 | 18 | 17 | 14 | 100 | 16 | 19 | 16 | 21 | 15 | 17 | 19 | 17 | 20 | 20 | 23 | 19 | 19 | 21 | 17 | 22 | 17 | 17 | 14 | 20 |
| 3EI3 | 16 | 16 | 17 | 15 | 18 | 18 | 19 | 18 | 17 | 18 | 20 | 16 | 20 | 16 | 100 | 18 | 17 | 18 | 18 | 17 | 19 | 18 | 18 | 15 | 17 | 20 | 16 | 19 | 21 | 18 | 18 | 15 | 15 | 21 |
| 3ODT | 21 | 17 | 18 | 18 | 18 | 17 | 19 | 17 | 18 | 20 | 14 | 19 | 20 | 19 | 18 | 100 | 18 | 26 | 20 | 20 | 19 | 18 | 27 | 18 | 22 | 20 | 16 | 21 | 24 | 19 | 18 | 20 | 16 | 23 |
| 2OVP | 28 | 17 | 19 | 20 | 17 | 16 | 15 | 18 | 17 | 31 | 19 | 18 | 18 | 16 | 17 | 18 | 100 | 22 | 19 | 18 | 17 | 14 | 23 | 15 | 19 | 19 | 18 | 22 | 20 | 19 | 16 | 19 | 17 | 24 |
| 3EMH | 23 | 19 | 22 | 19 | 19 | 20 | 19 | 17 | 19 | 19 | 18 | 17 | 17 | 21 | 18 | 26 | 22 | 100 | 16 | 21 | 19 | 21 | 32 | 17 | 25 | 22 | 20 | 25 | 24 | 25 | 19 | 18 | 20 | 27 |
| 3I2N | 15 | 18 | 17 | 17 | 20 | 17 | 17 | 17 | 16 | 15 | 17 | 16 | 17 | 15 | 18 | 20 | 19 | 16 | 100 | 19 | 21 | 15 | 18 | 19 | 17 | 17 | 16 | 19 | 19 | 17 | 19 | 19 | 17 | 20 |
| 3MMY | 15 | 16 | 15 | 19 | 21 | 16 | 19 | 18 | 16 | 17 | 15 | 16 | 17 | 17 | 17 | 20 | 18 | 21 | 19 | 100 | 16 | 17 | 16 | 21 | 20 | 16 | 15 | 17 | 19 | 14 | 16 | 16 | 15 | 18 |
| 1NR01 | 20 | 14 | 17 | 14 | 20 | 17 | 17 | 16 | 19 | 17 | 18 | 19 | 17 | 19 | 19 | 19 | 17 | 19 | 21 | 16 | 100 | 19 | 21 | 17 | 21 | 19 | 20 | 23 | 20 | 21 | 19 | 16 | 17 | 20 |
| 1NR02 | 18 | 19 | 18 | 16 | 16 | 19 | 20 | 17 | 15 | 15 | 15 | 17 | 16 | 17 | 18 | 18 | 14 | 21 | 14 | 17 | 19 | 100 | 18 | 19 | 23 | 17 | 17 | 15 | 17 | 19 | 16 | 19 | 15 | 18 |
| 1VYH | 23 | 19 | 21 | 21 | 20 | 17 | 23 | 16 | 19 | 20 | 17 | 16 | 17 | 21 | 18 | 27 | 23 | 32 | 18 | 16 | 21 | 18 | 100 | 16 | 21 | 20 | 20 | 24 | 27 | 23 | 19 | 20 | 14 | 24 |
| 1YFQ | 18 | 17 | 18 | 18 | 19 | 16 | 18 | 17 | 17 | 19 | 17 | 17 | 15 | 20 | 15 | 18 | 15 | 17 | 19 | 21 | 17 | 19 | 16 | 100 | 19 | 18 | 18 | 16 | 15 | 17 | 17 | 16 | 16 | 18 |
| 3OW8 | 18 | 22 | 22 | 15 | 23 | 19 | 18 | 18 | 19 | 18 | 20 | 16 | 19 | 23 | 17 | 22 | 19 | 25 | 17 | 20 | 21 | 23 | 21 | 19 | 100 | 20 | 17 | 21 | 16 | 18 | 15 | 16 | 17 | 23 |
| 4A11 | 19 | 14 | 20 | 18 | 17 | 17 | 21 | 17 | 20 | 16 | 17 | 16 | 18 | 19 | 20 | 20 | 19 | 22 | 17 | 16 | 19 | 17 | 20 | 18 | 20 | 100 | 17 | 18 | 19 | 18 | 17 | 17 | 16 | 21 |
| 4AEZ | 18 | 18 | 18 | 16 | 18 | 16 | 18 | 18 | 22 | 16 | 18 | 17 | 15 | 19 | 16 | 16 | 18 | 20 | 16 | 15 | 20 | 17 | 20 | 18 | 17 | 17 | 100 | 18 | 17 | 20 | 18 | 20 | 16 | 20 |
| 3MKQ | 20 | 21 | 21 | 18 | 15 | 19 | 16 | 15 | 17 | 16 | 17 | 17 | 17 | 21 | 18 | 21 | 22 | 25 | 19 | 17 | 23 | 15 | 24 | 16 | 21 | 18 | 18 | 100 | 22 | 20 | 19 | 18 | 18 | 22 |
| 3FRX | 20 | 18 | 17 | 18 | 17 | 20 | 17 | 15 | 17 | 19 | 20 | 17 | 16 | 17 | 21 | 24 | 20 | 24 | 19 | 19 | 20 | 17 | 27 | 15 | 16 | 19 | 17 | 22 | 100 | 20 | 19 | 18 | 15 | 21 |
| 1XHM | 18 | 19 | 19 | 20 | 16 | 17 | 23 | 19 | 16 | 19 | 19 | 16 | 17 | 22 | 18 | 19 | 19 | 25 | 17 | 14 | 21 | 19 | 23 | 17 | 18 | 18 | 20 | 20 | 20 | 100 | 19 | 19 | 16 | 22 |
| 3IIW | 18 | 15 | 18 | 18 | 15 | 17 | 18 | 17 | 18 | 19 | 17 | 18 | 17 | 17 | 18 | 18 | 16 | 19 | 19 | 16 | 19 | 16 | 19 | 17 | 15 | 17 | 18 | 19 | 19 | 19 | 100 | 17 | 16 | 16 |
| 1GXR | 19 | 18 | 16 | 20 | 19 | 17 | 18 | 19 | 18 | 15 | 18 | 17 | 16 | 17 | 18 | 20 | 19 | 18 | 19 | 16 | 16 | 19 | 20 | 16 | 16 | 17 | 20 | 18 | 18 | 19 | 17 | 100 | 19 | 26 |
| 3DW8 | 17 | 13 | 15 | 16 | 17 | 15 | 17 | 17 | 17 | 16 | 15 | 16 | 18 | 14 | 15 | 16 | 17 | 20 | 17 | 15 | 16 | 15 | 14 | 16 | 17 | 16 | 16 | 18 | 15 | 16 | 16 | 19 | 100 | 16 |
| 1ERJ | 23 | 16 | 21 | 19 | 20 | 18 | 20 | 19 | 18 | 22 | 16 | 15 | 19 | 20 | 21 | 23 | 24 | 27 | 20 | 18 | 20 | 18 | 24 | 18 | 23 | 21 | 20 | 22 | 21 | 22 | 16 | 26 | 16 | 100 |
