# Supplementary material for: A Method for WD40 Repeat Detection and Secondary Structure Prediction
Source: PLoS One. 2013 Jun 11;8(6):e65705. doi: 10.1371/journal.pone.0065705 (PMC3679165; doi:10.1371/journal.pone.0065705)
Supplement: Table S2 — Parameters used in function S1. (DOCX) [file pone.0065705.s006.docx]

The loop length distributions are fitted by the following procedure:

1. The fitting function used in the loop length score, where *l* is the loop length:

$$f\left( l \right)=kl^{p}[e^{\left( -l^{m} \right)}+e^{\left( -l^{n} \right)}]+w (\mathbf{S}\boldsymbol{1})$$

The parameters used in function S1 are listed in **Table S2**.

**Table S2**. Parameters used in function **S1**

|  | k | p | m | n | w |
| --- | --- | --- | --- | --- | --- |
| L_ab_ | 0.16699 | 4.82267 | 0.98221 | 1.10271 | -6.4467 |
| L_bc_ | 1.50144 | 2.91924 | 0.88315 | 1.15104 | -6.5384 |
| L_cd_ | 0.12488 | 4.73382 | 0.94705 | 1.07288 | -6.1084 |
| L_da_ | 0.00819 | 6.73036 | 1.01722 | 1.1012 | -6.2847 |
