# Supplementary material for: A Method for WD40 Repeat Detection and Secondary Structure Prediction
Source: PLoS One. 2013 Jun 11;8(6):e65705. doi: 10.1371/journal.pone.0065705 (PMC3679165; doi:10.1371/journal.pone.0065705)
Supplement: Table S3 — The final scores of loop length in the score function. (DOCX) [file pone.0065705.s007.docx]

1. Adjust the values in the curves by adding a linear penalty function. The final values embedded in the score function are list in **Table S3**.

**Table S3**. The final scores of loop length in the score function.

| **length** | **2** | **3** | **4** | **5** | **6** | **7** | **8** |
| --- | --- | --- | --- | --- | --- | --- | --- |
| **L_ab_** | 1.21 | 2.92 | 4.03 | 4.12 | 3.52 | 2.68 | 1.89 |
| **L_bc_** | 3.03 | 3.73 | 3.48 | 2.90 | 2.27 | 1.70 | 1.24 |
| **L_cd_** | 0.89 | 2.21 | 3.21 | 3.50 | 3.22 | 2.66 | 2.03 |
| **L_da_** | 0.22 | 1.09 | 2.46 | 3.58 | 3.97 | 3.66 | 2.96 |
| **length** | **9** | **10** | **11** | **12** | **13** | **14** | **15** |
| **L_ab_** | 1.25 | 0.79 | 0.33 | -0.12 | -0.49 | -0.81 | -1.10 |
| **L_bc_** | 0.87 | 0.40 | -0.05 | -0.43 | -0.77 | -1.09 | -1.38 |
| **L_cd_** | 1.47 | 1.02 | 0.60 | 0.04 | -0.44 | -0.87 | -1.25 |
| **L_da_** | 2.17 | 1.48 | 0.74 | 0.06 | -0.49 | -0.95 | -1.35 |
| **length** | **16** | **17** | **18** | **19** | **20** | **21** | **22** |
| **L_ab_** | -1.37 | -1.64 | -1.89 | -2.15 | -2.40 | -2.65 | -2.90 |
| **L_bc_** | -1.65 | -1.92 | -2.18 | -2.44 | -2.69 | -2.95 | -3.20 |
| **L_cd_** | -1.62 | -1.96 | -2.30 | -2.63 | -2.95 | -3.28 | -3.60 |
| **L_da_** | -1.71 | -2.04 | -2.37 | -2.68 | -3.00 | -3.31 | -3.62 |
| **length** | **23** | **24** | **25** | **26** | **27** | **28** | **29** |
| **L_ab_** | -3.15 | -3.40 | -3.65 | -3.90 | -4.15 | -4.40 | -4.65 |
| **L_bc_** | -3.45 | -3.70 | -3.95 | -4.20 | -4.45 | -4.70 | -4.95 |
| **L_cd_** | -3.92 | -4.24 | -4.56 | -4.88 | -5.20 | -5.52 | -5.84 |
| **L_da_** | -3.93 | -4.24 | -4.55 | -4.86 | -5.17 | -5.48 | -5.79 |
