# Supplementary material for: A Method for WD40 Repeat Detection and Secondary Structure Prediction
Source: PLoS One. 2013 Jun 11;8(6):e65705. doi: 10.1371/journal.pone.0065705 (PMC3679165; doi:10.1371/journal.pone.0065705)
Supplement: Table S4 — Residue frequencies of every position in the sequence logo. (DOCX) [file pone.0065705.s008.docx]

**Table S4**. Residue frequencies of every position in the sequence logo.

|  | **G** | **A** | **P** | **V** | **I** | **L** | **F** | **C** | **M** | **W** | **Y** | **Q** | **T** | **S** | **N** | **H** | **D** | **E** | **K** | **R** |
| --- | --- | --- | --- | --- | --- | --- | --- | --- | --- | --- | --- | --- | --- | --- | --- | --- | --- | --- | --- | --- |
| **Sd1** | 0.013 | 0.050 | 0.017 | 0.025 | 0.042 | 0.025 | 0.017 | 0.013 | 0.017 | 0.008 | 0.013 | 0.076 | 0.097 | 0.092 | 0.059 | 0.017 | 0.038 | 0.151 | 0.189 | 0.042 |
| **Sd2** | 0.013 | 0.038 | 0.105 | 0.071 | 0.063 | 0.214 | 0.046 | 0.147 | 0.004 | 0.000 | 0.017 | 0.042 | 0.034 | 0.025 | 0.021 | 0.000 | 0.008 | 0.050 | 0.063 | 0.038 |
| **Sd3** | 0.008 | 0.038 | 0.008 | 0.172 | 0.134 | 0.197 | 0.021 | 0.004 | 0.038 | 0.000 | 0.025 | 0.038 | 0.063 | 0.050 | 0.008 | 0.017 | 0.025 | 0.042 | 0.071 | 0.038 |
| **Sd4** | 0.071 | 0.134 | 0.013 | 0.025 | 0.034 | 0.034 | 0.067 | 0.008 | 0.017 | 0.008 | 0.034 | 0.080 | 0.059 | 0.067 | 0.034 | 0.071 | 0.025 | 0.021 | 0.097 | 0.101 |
| **Sd5** | 0.013 | 0.017 | 0.000 | 0.092 | 0.084 | 0.076 | 0.013 | 0.004 | 0.017 | 0.004 | 0.004 | 0.050 | 0.282 | 0.076 | 0.046 | 0.021 | 0.021 | 0.084 | 0.050 | 0.046 |
| **Sd6** | 0.013 | 0.021 | 0.004 | 0.046 | 0.092 | 0.357 | 0.151 | 0.000 | 0.017 | 0.013 | 0.042 | 0.017 | 0.042 | 0.021 | 0.013 | 0.025 | 0.025 | 0.025 | 0.059 | 0.017 |
| **Lda1** | 0.025 | 0.044 | 0.031 | 0.044 | 0.063 | 0.038 | 0.025 | 0.013 | 0.019 | 0.000 | 0.025 | 0.094 | 0.113 | 0.088 | 0.063 | 0.025 | 0.069 | 0.100 | 0.088 | 0.038 |
| **Lda2** | 0.356 | 0.105 | 0.025 | 0.050 | 0.017 | 0.042 | 0.017 | 0.008 | 0.008 | 0.004 | 0.000 | 0.033 | 0.029 | 0.054 | 0.067 | 0.004 | 0.054 | 0.071 | 0.042 | 0.013 |
| **Lda3** | 0.021 | 0.033 | 0.025 | 0.013 | 0.050 | 0.038 | 0.046 | 0.013 | 0.000 | 0.004 | 0.008 | 0.008 | 0.017 | 0.042 | 0.025 | 0.565 | 0.033 | 0.021 | 0.017 | 0.021 |
| **Lda4** | 0.054 | 0.054 | 0.033 | 0.021 | 0.021 | 0.029 | 0.004 | 0.008 | 0.013 | 0.000 | 0.013 | 0.042 | 0.126 | 0.117 | 0.079 | 0.008 | 0.096 | 0.105 | 0.117 | 0.059 |
| **Lda5** | 0.121 | 0.092 | 0.017 | 0.017 | 0.013 | 0.021 | 0.008 | 0.013 | 0.008 | 0.004 | 0.042 | 0.046 | 0.029 | 0.138 | 0.067 | 0.046 | 0.146 | 0.063 | 0.067 | 0.042 |
| **Lda6** | 0.075 | 0.079 | 0.130 | 0.042 | 0.017 | 0.033 | 0.042 | 0.004 | 0.008 | 0.050 | 0.046 | 0.054 | 0.054 | 0.113 | 0.033 | 0.013 | 0.046 | 0.063 | 0.046 | 0.050 |
| **Sa1** | 0.013 | 0.034 | 0.008 | 0.492 | 0.290 | 0.063 | 0.017 | 0.025 | 0.004 | 0.000 | 0.013 | 0.008 | 0.008 | 0.013 | 0.004 | 0.004 | 0.000 | 0.000 | 0.004 | 0.000 |
| **Sa2** | 0.017 | 0.013 | 0.000 | 0.038 | 0.021 | 0.097 | 0.063 | 0.034 | 0.025 | 0.080 | 0.063 | 0.013 | 0.118 | 0.113 | 0.118 | 0.025 | 0.008 | 0.013 | 0.042 | 0.101 |
| **Sa3** | 0.076 | 0.089 | 0.004 | 0.030 | 0.004 | 0.017 | 0.013 | 0.144 | 0.004 | 0.000 | 0.008 | 0.030 | 0.072 | 0.288 | 0.021 | 0.025 | 0.102 | 0.008 | 0.025 | 0.038 |
| **Sa4** | 0.013 | 0.075 | 0.000 | 0.356 | 0.142 | 0.238 | 0.033 | 0.054 | 0.029 | 0.000 | 0.000 | 0.000 | 0.021 | 0.021 | 0.004 | 0.008 | 0.004 | 0.000 | 0.000 | 0.000 |
| **Sa5** | 0.021 | 0.192 | 0.000 | 0.029 | 0.029 | 0.008 | 0.008 | 0.046 | 0.000 | 0.008 | 0.013 | 0.067 | 0.046 | 0.126 | 0.017 | 0.025 | 0.134 | 0.046 | 0.121 | 0.063 |
| **Sa6** | 0.008 | 0.021 | 0.004 | 0.075 | 0.075 | 0.059 | 0.301 | 0.021 | 0.013 | 0.276 | 0.084 | 0.000 | 0.033 | 0.008 | 0.004 | 0.004 | 0.004 | 0.000 | 0.008 | 0.000 |
| **Lab1** | 0.031 | 0.071 | 0.009 | 0.035 | 0.040 | 0.053 | 0.022 | 0.031 | 0.018 | 0.000 | 0.013 | 0.009 | 0.049 | 0.292 | 0.142 | 0.093 | 0.066 | 0.004 | 0.009 | 0.013 |
| **Lab2** | 0.044 | 0.053 | 0.396 | 0.018 | 0.000 | 0.027 | 0.013 | 0.013 | 0.000 | 0.004 | 0.018 | 0.031 | 0.018 | 0.067 | 0.049 | 0.053 | 0.071 | 0.027 | 0.067 | 0.031 |
| **Lab3** | 0.046 | 0.033 | 0.021 | 0.017 | 0.004 | 0.029 | 0.013 | 0.013 | 0.004 | 0.008 | 0.017 | 0.029 | 0.084 | 0.088 | 0.138 | 0.042 | 0.280 | 0.033 | 0.059 | 0.042 |
| **Lab4** | 0.339 | 0.018 | 0.009 | 0.009 | 0.009 | 0.032 | 0.014 | 0.009 | 0.000 | 0.000 | 0.023 | 0.023 | 0.037 | 0.110 | 0.073 | 0.023 | 0.092 | 0.078 | 0.073 | 0.028 |
| **Lab5** | 0.034 | 0.034 | 0.069 | 0.015 | 0.005 | 0.030 | 0.020 | 0.015 | 0.005 | 0.005 | 0.025 | 0.064 | 0.084 | 0.113 | 0.128 | 0.010 | 0.034 | 0.099 | 0.133 | 0.079 |
| **Sb1** | 0.008 | 0.021 | 0.004 | 0.071 | 0.113 | 0.172 | 0.076 | 0.004 | 0.021 | 0.029 | 0.118 | 0.029 | 0.055 | 0.021 | 0.004 | 0.038 | 0.017 | 0.025 | 0.092 | 0.080 |
| **Sb2** | 0.000 | 0.017 | 0.000 | 0.163 | 0.243 | 0.385 | 0.113 | 0.021 | 0.033 | 0.000 | 0.017 | 0.000 | 0.004 | 0.000 | 0.000 | 0.000 | 0.000 | 0.000 | 0.004 | 0.000 |
| **Sb3** | 0.004 | 0.331 | 0.000 | 0.230 | 0.130 | 0.134 | 0.042 | 0.033 | 0.021 | 0.004 | 0.033 | 0.008 | 0.008 | 0.021 | 0.000 | 0.000 | 0.000 | 0.000 | 0.000 | 0.000 |
| **Sb4** | 0.004 | 0.109 | 0.000 | 0.100 | 0.021 | 0.033 | 0.008 | 0.029 | 0.000 | 0.004 | 0.013 | 0.000 | 0.259 | 0.410 | 0.000 | 0.000 | 0.000 | 0.004 | 0.004 | 0.000 |
| **Sb5** | 0.402 | 0.230 | 0.008 | 0.071 | 0.017 | 0.013 | 0.004 | 0.084 | 0.008 | 0.000 | 0.000 | 0.000 | 0.054 | 0.096 | 0.000 | 0.000 | 0.000 | 0.000 | 0.008 | 0.004 |
| **Sb6** | 0.205 | 0.050 | 0.004 | 0.017 | 0.004 | 0.029 | 0.017 | 0.038 | 0.013 | 0.000 | 0.025 | 0.000 | 0.050 | 0.456 | 0.013 | 0.021 | 0.054 | 0.004 | 0.000 | 0.000 |
| **Lbc1** | 0.082 | 0.073 | 0.013 | 0.030 | 0.017 | 0.056 | 0.030 | 0.022 | 0.026 | 0.039 | 0.047 | 0.034 | 0.043 | 0.103 | 0.047 | 0.017 | 0.103 | 0.069 | 0.069 | 0.078 |
| **Lbc2** | 0.017 | 0.017 | 0.000 | 0.004 | 0.000 | 0.013 | 0.013 | 0.000 | 0.000 | 0.000 | 0.000 | 0.008 | 0.021 | 0.046 | 0.092 | 0.004 | 0.674 | 0.046 | 0.029 | 0.017 |
| **Lbc3** | 0.296 | 0.030 | 0.004 | 0.009 | 0.000 | 0.013 | 0.026 | 0.022 | 0.009 | 0.000 | 0.004 | 0.030 | 0.022 | 0.083 | 0.143 | 0.017 | 0.035 | 0.022 | 0.130 | 0.104 |
| **Sc1** | 0.029 | 0.029 | 0.000 | 0.042 | 0.042 | 0.050 | 0.029 | 0.013 | 0.021 | 0.000 | 0.013 | 0.029 | 0.294 | 0.105 | 0.055 | 0.017 | 0.034 | 0.042 | 0.097 | 0.059 |
| **Sc2** | 0.008 | 0.050 | 0.004 | 0.293 | 0.339 | 0.184 | 0.029 | 0.033 | 0.013 | 0.000 | 0.004 | 0.004 | 0.017 | 0.013 | 0.000 | 0.004 | 0.000 | 0.000 | 0.004 | 0.000 |
| **Sc3** | 0.000 | 0.054 | 0.000 | 0.017 | 0.067 | 0.067 | 0.029 | 0.013 | 0.013 | 0.008 | 0.033 | 0.029 | 0.033 | 0.059 | 0.054 | 0.042 | 0.008 | 0.025 | 0.234 | 0.213 |
| **Sc4** | 0.008 | 0.021 | 0.004 | 0.297 | 0.268 | 0.238 | 0.050 | 0.013 | 0.017 | 0.008 | 0.008 | 0.004 | 0.008 | 0.021 | 0.000 | 0.004 | 0.000 | 0.004 | 0.021 | 0.004 |
| **Sc5** | 0.000 | 0.008 | 0.000 | 0.042 | 0.017 | 0.042 | 0.100 | 0.008 | 0.004 | 0.582 | 0.151 | 0.008 | 0.000 | 0.004 | 0.000 | 0.021 | 0.000 | 0.004 | 0.000 | 0.008 |
| **Sc6** | 0.013 | 0.008 | 0.013 | 0.013 | 0.004 | 0.008 | 0.004 | 0.000 | 0.000 | 0.000 | 0.013 | 0.038 | 0.025 | 0.075 | 0.138 | 0.021 | 0.414 | 0.096 | 0.084 | 0.033 |
| **Lcd1** | 0.017 | 0.057 | 0.040 | 0.190 | 0.098 | 0.241 | 0.034 | 0.011 | 0.011 | 0.017 | 0.057 | 0.011 | 0.052 | 0.057 | 0.006 | 0.006 | 0.006 | 0.023 | 0.052 | 0.011 |
| **Lcd2** | 0.058 | 0.069 | 0.035 | 0.029 | 0.035 | 0.012 | 0.017 | 0.000 | 0.006 | 0.000 | 0.035 | 0.064 | 0.023 | 0.104 | 0.075 | 0.017 | 0.069 | 0.150 | 0.110 | 0.092 |
| **Lcd3** | 0.059 | 0.053 | 0.012 | 0.006 | 0.006 | 0.018 | 0.018 | 0.012 | 0.006 | 0.000 | 0.012 | 0.035 | 0.265 | 0.147 | 0.135 | 0.018 | 0.076 | 0.035 | 0.076 | 0.012 |
