# Supplementary material for: A Method for WD40 Repeat Detection and Secondary Structure Prediction
Source: PLoS One. 2013 Jun 11;8(6):e65705. doi: 10.1371/journal.pone.0065705 (PMC3679165; doi:10.1371/journal.pone.0065705)
Supplement: Table S5 — Repeat detection comparison by different methods. (DOCX) [file pone.0065705.s009.docx]

**Table S5**. Repeat detection comparison by different methods.

| Num | ID | Name | PDB | UNIPROT | | SMART | | PFAM | | PROSITE | | WDSP | |
| --- | --- | --- | --- | --- | --- | --- | --- | --- | --- | --- | --- | --- | --- |
|  |  |  | abcd | abc | abcd | abc | abcd | abc | abcd | abc | abcd | abc | abcd |
| 1 | 1ERJ | TUP1 | 7 | 7 | 0 | 7 | 6 | 7 | 5 | 6 | 4 | 7 | 5 |
| 2 | 1GXR | TLE1 | 7 | 6 | 0 | 7 | 7 | 6 | 5 | 2 | 2 | 7 | 7 |
| 3 | 1K8K | ARPC1 | 7 | 6 | 0 | 4 | 3 | 3 | 3 | 1 | 0 | 7 | 6 |
| 4 | 1NEX | CDC4 | 8 | 7 | 0 | 7 | 7 | 6 | 6 | 5 | 3 | 8 | 8 |
| 5 | 1NR0 | AIP1 | 14 | 9 | 6 | 10 | 10 | 7 | 4 | 6 | 2 | 13 | 12 |
| 6 | 1P22 | β-TrCP | 7 | 7 | 2 | 7 | 7 | 7 | 6 | 6 | 3 | 7 | 7 |
| 7 | 1R5M | SIF2 | 8 | 8 | 1 | 5 | 4 | 4 | 2 | 3 | 2 | 7 | 5 |
| 8 | 1SQ9 | SKI8P | 7 | 7 | 0 | 4 | 1 | 2 | 0 | 1 | 0 | 7 | 3 |
| 9 | 1VYH | LIS1 | 7 | 7 | 6 | 7 | 7 | 7 | 7 | 7 | 5 | 7 | 7 |
| 10 | 1XHM | Gβ | 7 | 7 | 0 | 7 | 7 | 7 | 5 | 6 | 4 | 6 | 6 |
| 11 | 1YFQ | BUB3 | 7 | 6 | 2 | 4 | 4 | 2 | 1 | 1 | 1 | 7 | 7 |
| 12 | 2AQ5 | coronin-1 | 7 | 7 | 7 | 3 | 3 | 3 | 1 | 2 | 1 | 6 | 5 |
| 13 | 2HES | CIA1 | 7 | 7 | 1 | 7 | 6 | 7 | 5 | 4 | 0 | 7 | 7 |
| 14 | 2VDU | Trm82 | 7 | 5 | 5 | 3 | 2 | 1 | 0 | 1 | 0 | 7 | 5 |
| 15 | 2PM9 | Sec31 | 7 | 7 | 3 | 6 | 5 | 3 | 2 | 3 | 1 | 7 | 5 |
| 16 | 3ACP | Rpn14 | 7 | 6 | 4 | 3 | 3 | 2 | 1 | 2 | 2 | 7 | 7 |
| 17 | 3DW8 | Phosphatase | 7 | 7 | 2 | 7 | 1 | 2 | 0 | 0 | 0 | 7 | 2 |
| 18 | 3EI3 | DDB2 | 7 | 5 | 2 | 5 | 5 | 2 | 1 | 1 | 0 | 7 | 7 |
| 19 | 3EMH | WDR5 | 7 | 7 | 6 | 7 | 7 | 7 | 7 | 6 | 6 | 7 | 7 |
| 20 | 3EWE | Seh1 | 6 | 5 | 0 | 5 | 3 | 5 | 3 | 3 | 0 | 6 | 5 |
| 21 | 3FRX | ASC1 | 7 | 7 | 5 | 7 | 6 | 6 | 6 | 5 | 2 | 7 | 6 |
| 22 | 3GFC | RbBP4 | 7 | 6 | 0 | 6 | 6 | 5 | 5 | 5 | 0 | 7 | 6 |
| 23 | 3I2N | WDR92 | 7 | 6 | 3 | 4 | 4 | 2 | 1 | 0 | 0 | 7 | 7 |
| 24 | 3IIW | EED | 7 | 7 | 2 | 6 | 6 | 2 | 1 | 2 | 1 | 7 | 7 |
| 25 | 3JRP | Sec13 | 6 | 6 | 1 | 6 | 6 | 5 | 5 | 1 | 0 | 6 | 6 |
| 26 | 3MMY | Rae1 | 7 | 4 | 0 | 4 | 4 | 4 | 2 | 3 | 3 | 6 | 6 |
| 27 | 3ODT | Doa1 | 7 | 7 | 0 | 6 | 6 | 5 | 5 | 4 | 1 | 7 | 7 |
| 28 | 3MKQ | Cop1 | 7 | 6 | 0 | 7 | 7 | 5 | 5 | 4 | 4 | 7 | 7 |
| 29 | 3OW8 | WDR61 | 7 | 7 | 5 | 7 | 7 | 7 | 5 | 6 | 4 | 7 | 7 |
| 30 | 2OVP | FBXW7 | 8 | 7 | 7 | 8 | 8 | 7 | 7 | 7 | 7 | 8 | 7 |
| 31 | 4A11 | CSA | 7 | 5 | 0 | 5 | 3 | 5 | 2 | 5 | 0 | 7 | 4 |
| 32 | 3ZWL | EIF3B | 7 | 5 | 3 | 6 | 6 | 4 | 3 | 3 | 2 | 7 | 7 |
| 33 | 4AEZ | SLP1 | 7 | 7 | 4 | 6 | 6 | 3 | 3 | 2 | 0 | 7 | 7 |
|  | Total |  | 239 | 213 | 77 | 193 | 173 | 150 | 114 | 113 | 60 | 234 | 207 |
|  | Percentage% | |  | 89.1 | 32.2 | 80.8 | 72.4 | 62.8 | 47.7 | 47.3 | 25.1 | 97.9 | 86.6 |
