# Supplementary material for: A Method for WD40 Repeat Detection and Secondary Structure Prediction
Source: PLoS One. 2013 Jun 11;8(6):e65705. doi: 10.1371/journal.pone.0065705 (PMC3679165; doi:10.1371/journal.pone.0065705)
Supplement: Table S6 — 76 potential WD40 proteins are only detected by WDSP. (DOCX) [file pone.0065705.s010.docx]

**Table S6**. 76 potential WD40 proteins are only detected by WDSP. 35 of them are in agreement with InterPro, SUPERFAMILY or Gene3D annotation. Other 36 of them are β-propeller proteins. Little annotation is available for the rest 5 proteins.

|  | WD40 | | | | Other repeats | | | | | | | | | WDSP^*^ | | | | | | |
| --- | --- | --- | --- | --- | --- | --- | --- | --- | --- | --- | --- | --- | --- | --- | --- | --- | --- | --- | --- | --- |
| ID | Gene3D | InterPro | SUPFAM | **sum** | Kelch | TolB | RCC1 | PQQ | PbH1 | Quinonprotein_ADH | MAM | VPS_repeat | **sum** | Repeat | WDSP_d | WDSP_p | avg. score | pentad | tetrad | traid |
| FB122_ARATH | 0 | 0 | 0 | 0 | 0 | 0 | 0 | 0 | 0 | 0 | 0 | 0 | 0 | 6 | 1 | 1 | 48 | 0 | 0 | 0 |
| NOL11_XENLA | 0 | 0 | 0 | 0 | 0 | 0 | 0 | 0 | 0 | 0 | 0 | 0 | 0 | 7 | 1 | 1 | 51 | 0 | 0 | 0 |
| POF12_SCHPO | 0 | 0 | 0 | 0 | 0 | 0 | 0 | 0 | 0 | 0 | 0 | 0 | 0 | 7 | 1 | 1 | 48 | 0 | 0 | 1 |
| VPS16_YEAST | 0 | 0 | 0 | 0 | 0 | 0 | 0 | 0 | 0 | 0 | 0 | 0 | 0 | 7 | 1 | 1 | 50 | 0 | 0 | 0 |
| YAG9_SCHPO | 0 | 0 | 0 | 0 | 0 | 0 | 0 | 0 | 0 | 0 | 0 | 0 | 0 | 7 | 1 | 1 | 49 | 0 | 0 | 0 |
| 6PGL_BUCAP | 1 | 1 | 0 | 2 | 0 | 0 | 0 | 0 | 0 | 0 | 0 | 0 | 0 | 6 | 1 | 1 | 62 | 0 | 0 | 0 |
| DDB1_DICDI | 0 | 1 | 1 | 2 | 0 | 0 | 0 | 0 | 0 | 0 | 0 | 0 | 0 | 20 | 3 | 1 | 49 | 0 | 0 | 0 |
| EIF3B_ARATH | 1 | 1 | 0 | 2 | 0 | 0 | 0 | 0 | 0 | 0 | 0 | 0 | 0 | 9 | 2 | 1 | 60 | 0 | 0 | 1 |
| EIF3B_TOBAC | 1 | 1 | 0 | 2 | 0 | 0 | 0 | 0 | 0 | 0 | 0 | 0 | 0 | 7 | 1 | 1 | 51 | 0 | 0 | 0 |
| ELP1_SCHPO | 1 | 1 | 0 | 2 | 0 | 0 | 0 | 0 | 0 | 0 | 0 | 0 | 0 | 12 | 2 | 1 | 50 | 0 | 0 | 0 |
| NIRF_PSEST | 1 | 1 | 0 | 2 | 0 | 0 | 0 | 0 | 0 | 0 | 0 | 0 | 0 | 7 | 1 | 1 | 50 | 0 | 0 | 0 |
| NSA1_CANGA | 1 | 1 | 0 | 2 | 0 | 0 | 0 | 0 | 0 | 0 | 0 | 0 | 0 | 7 | 1 | 1 | 48 | 0 | 0 | 0 |
| VPS11_CAEEL | 1 | 1 | 0 | 2 | 0 | 0 | 0 | 0 | 0 | 0 | 0 | 0 | 0 | 6 | 1 | 1 | 52 | 0 | 0 | 0 |
| Y4094_ARATH | 1 | 1 | 0 | 2 | 0 | 0 | 0 | 0 | 0 | 0 | 0 | 0 | 0 | 7 | 1 | 1 | 53 | 0 | 0 | 1 |
| Y598_TREPA | 1 | 1 | 0 | 2 | 0 | 0 | 0 | 0 | 0 | 0 | 0 | 0 | 0 | 10 | 2 | 1 | 51 | 0 | 0 | 1 |
| YKL1_SCHPO | 1 | 1 | 0 | 2 | 0 | 0 | 0 | 0 | 0 | 0 | 0 | 0 | 0 | 18 | 3 | 1 | 54 | 0 | 0 | 0 |
| BBS2_DANRE | 1 | 1 | 1 | 3 | 0 | 0 | 0 | 0 | 0 | 0 | 0 | 0 | 0 | 7 | 1 | 1 | 57 | 0 | 0 | 0 |
| BBS2_HUMAN | 1 | 1 | 1 | 3 | 0 | 0 | 0 | 0 | 0 | 0 | 0 | 0 | 0 | 7 | 1 | 1 | 57 | 0 | 0 | 0 |
| BBS2_MOUSE | 1 | 1 | 1 | 3 | 0 | 0 | 0 | 0 | 0 | 0 | 0 | 0 | 0 | 7 | 1 | 1 | 58 | 0 | 0 | 0 |
| BBS2_RAT | 1 | 1 | 1 | 3 | 0 | 0 | 0 | 0 | 0 | 0 | 0 | 0 | 0 | 7 | 1 | 1 | 57 | 0 | 0 | 0 |
| DCAF1_DROME | 1 | 1 | 1 | 3 | 0 | 0 | 0 | 0 | 0 | 0 | 0 | 0 | 0 | 6 | 1 | 1 | 55 | 0 | 0 | 1 |
| FAR8_YEAST | 1 | 1 | 1 | 3 | 0 | 0 | 0 | 0 | 0 | 0 | 0 | 0 | 0 | 7 | 1 | 1 | 54 | 0 | 0 | 0 |
| HPS5_HUMAN | 1 | 1 | 1 | 3 | 0 | 0 | 0 | 0 | 0 | 0 | 0 | 0 | 0 | 7 | 1 | 1 | 59 | 0 | 0 | 0 |
| HPS5_MOUSE | 1 | 1 | 1 | 3 | 0 | 0 | 0 | 0 | 0 | 0 | 0 | 0 | 0 | 7 | 1 | 1 | 58 | 0 | 0 | 0 |
| NSA1_CANAL | 1 | 1 | 1 | 3 | 0 | 0 | 0 | 0 | 0 | 0 | 0 | 0 | 0 | 7 | 1 | 1 | 57 | 0 | 0 | 0 |
| NSA1_DEBHA | 1 | 1 | 1 | 3 | 0 | 0 | 0 | 0 | 0 | 0 | 0 | 0 | 0 | 7 | 1 | 1 | 59 | 0 | 0 | 0 |
| NSA1_LODEL | 1 | 1 | 1 | 3 | 0 | 0 | 0 | 0 | 0 | 0 | 0 | 0 | 0 | 7 | 1 | 1 | 50 | 0 | 0 | 0 |
| NSA1_PICST | 1 | 1 | 1 | 3 | 0 | 0 | 0 | 0 | 0 | 0 | 0 | 0 | 0 | 6 | 1 | 1 | 56 | 0 | 0 | 0 |
| PAN2_CHICK | 1 | 1 | 1 | 3 | 0 | 0 | 0 | 0 | 0 | 0 | 0 | 0 | 0 | 7 | 1 | 1 | 61 | 0 | 0 | 0 |
| PEP5_SCHPO | 1 | 1 | 1 | 3 | 0 | 0 | 0 | 0 | 0 | 0 | 0 | 0 | 0 | 6 | 1 | 1 | 51 | 0 | 0 | 0 |
| RAV1_SCHPO | 1 | 1 | 1 | 3 | 0 | 0 | 0 | 0 | 0 | 0 | 0 | 0 | 0 | 14 | 2 | 1 | 50 | 0 | 1 | 0 |
| SAP_ARATH | 1 | 1 | 1 | 3 | 0 | 0 | 0 | 0 | 0 | 0 | 0 | 0 | 0 | 7 | 1 | 1 | 51 | 0 | 0 | 0 |
| SWT21_KLULA | 1 | 1 | 1 | 3 | 0 | 0 | 0 | 0 | 0 | 0 | 0 | 0 | 0 | 7 | 1 | 1 | 53 | 0 | 0 | 0 |
| SWT21_VANPO | 1 | 1 | 1 | 3 | 0 | 0 | 0 | 0 | 0 | 0 | 0 | 0 | 0 | 6 | 1 | 1 | 48 | 0 | 0 | 0 |
| SWT21_YEAS2 | 1 | 1 | 1 | 3 | 0 | 0 | 0 | 0 | 0 | 0 | 0 | 0 | 0 | 7 | 1 | 1 | 50 | 0 | 0 | 0 |
| VPS11_HUMAN | 1 | 1 | 1 | 3 | 0 | 0 | 0 | 0 | 0 | 0 | 0 | 0 | 0 | 7 | 1 | 1 | 53 | 0 | 0 | 0 |
| VPS11_MOUSE | 1 | 1 | 1 | 3 | 0 | 0 | 0 | 0 | 0 | 0 | 0 | 0 | 0 | 7 | 1 | 1 | 52 | 0 | 0 | 0 |
| VPS41_HUMAN | 1 | 1 | 1 | 3 | 0 | 0 | 0 | 0 | 0 | 0 | 0 | 0 | 0 | 7 | 1 | 1 | 56 | 0 | 0 | 0 |
| VPS41_MOUSE | 1 | 1 | 1 | 3 | 0 | 0 | 0 | 0 | 0 | 0 | 0 | 0 | 0 | 7 | 1 | 1 | 56 | 0 | 0 | 0 |
| YL788_MIMIV | 1 | 1 | 1 | 3 | 0 | 0 | 0 | 0 | 0 | 0 | 0 | 0 | 0 | 6 | 1 | 1 | 48 | 0 | 0 | 0 |
| ATRN_MOUSE | 0 | 0 | 0 | 0 | 1 | 0 | 0 | 0 | 0 | 0 | 0 | 0 | 1 | 6 | 1 | 1 | 48 | 0 | 0 | 0 |
| CALI_HUMAN | 0 | 0 | 0 | 0 | 1 | 0 | 0 | 0 | 0 | 0 | 0 | 0 | 1 | 6 | 1 | 1 | 49 | 0 | 0 | 0 |
| CALI_MOUSE | 0 | 0 | 0 | 0 | 1 | 0 | 0 | 0 | 0 | 0 | 0 | 0 | 1 | 6 | 1 | 1 | 48 | 0 | 0 | 0 |
| CGLA_ALTCA | 0 | 0 | 0 | 0 | 0 | 0 | 0 | 0 | 0 | 1 | 0 | 0 | 1 | 6 | 1 | 1 | 49 | 0 | 0 | 0 |
| CGLA_PSEAS | 0 | 0 | 0 | 0 | 0 | 0 | 0 | 0 | 0 | 1 | 0 | 0 | 1 | 6 | 1 | 1 | 48 | 0 | 0 | 0 |
| ELP1_MOUSE | 0 | 0 | 0 | 0 | 0 | 1 | 0 | 0 | 0 | 0 | 0 | 0 | 1 | 7 | 1 | 1 | 56 | 0 | 0 | 0 |
| ENC1_HUMAN | 0 | 0 | 0 | 0 | 1 | 0 | 0 | 0 | 0 | 0 | 0 | 0 | 1 | 6 | 1 | 1 | 59 | 0 | 0 | 0 |
| ENC1_MOUSE | 0 | 0 | 0 | 0 | 1 | 0 | 0 | 0 | 0 | 0 | 0 | 0 | 1 | 6 | 1 | 1 | 60 | 0 | 0 | 1 |
| FBK28_ARATH | 0 | 0 | 0 | 0 | 1 | 0 | 0 | 0 | 0 | 0 | 0 | 0 | 1 | 6 | 1 | 1 | 50 | 0 | 0 | 0 |
| FBK61_ARATH | 0 | 0 | 0 | 0 | 1 | 0 | 0 | 0 | 0 | 0 | 0 | 0 | 1 | 6 | 1 | 1 | 49 | 0 | 0 | 0 |
| FBK67_ARATH | 0 | 0 | 0 | 0 | 1 | 0 | 0 | 0 | 0 | 0 | 0 | 0 | 1 | 6 | 1 | 1 | 48 | 0 | 0 | 0 |
| GACGG_DICDI | 0 | 0 | 0 | 0 | 0 | 0 | 1 | 0 | 0 | 0 | 0 | 0 | 1 | 6 | 1 | 1 | 58 | 0 | 0 | 0 |
| HERC3_HUMAN | 0 | 0 | 0 | 0 | 0 | 0 | 1 | 0 | 0 | 0 | 0 | 0 | 1 | 7 | 1 | 1 | 48 | 0 | 0 | 0 |
| KEL10_CAEEL | 0 | 0 | 0 | 0 | 1 | 0 | 0 | 0 | 0 | 0 | 0 | 0 | 1 | 10 | 2 | 1 | 48 | 0 | 0 | 0 |
| KLD8B_BOVIN | 0 | 0 | 0 | 0 | 1 | 0 | 0 | 0 | 0 | 0 | 0 | 0 | 1 | 7 | 1 | 1 | 54 | 0 | 0 | 0 |
| KLD8B_HUMAN | 0 | 0 | 0 | 0 | 1 | 0 | 0 | 0 | 0 | 0 | 0 | 0 | 1 | 7 | 1 | 1 | 54 | 0 | 0 | 0 |
| KLD8B_MOUSE | 0 | 0 | 0 | 0 | 1 | 0 | 0 | 0 | 0 | 0 | 0 | 0 | 1 | 7 | 1 | 1 | 54 | 0 | 0 | 0 |
| KLD8B_PONAB | 0 | 0 | 0 | 0 | 1 | 0 | 0 | 0 | 0 | 0 | 0 | 0 | 1 | 7 | 1 | 1 | 54 | 0 | 0 | 0 |
| KLD8B_RAT | 0 | 0 | 0 | 0 | 1 | 0 | 0 | 0 | 0 | 0 | 0 | 0 | 1 | 7 | 1 | 1 | 54 | 0 | 0 | 0 |
| KLH10_HUMAN | 0 | 0 | 0 | 0 | 1 | 0 | 0 | 0 | 0 | 0 | 0 | 0 | 1 | 6 | 1 | 1 | 48 | 0 | 0 | 0 |
| KLH14_CHICK | 0 | 0 | 0 | 0 | 1 | 0 | 0 | 0 | 0 | 0 | 0 | 0 | 1 | 6 | 1 | 1 | 56 | 0 | 0 | 0 |
| KLH14_MOUSE | 0 | 0 | 0 | 0 | 1 | 0 | 0 | 0 | 0 | 0 | 0 | 0 | 1 | 6 | 1 | 1 | 55 | 0 | 0 | 0 |
| KLH38_HUMAN | 0 | 0 | 0 | 0 | 1 | 0 | 0 | 0 | 0 | 0 | 0 | 0 | 1 | 6 | 1 | 1 | 51 | 0 | 1 | 0 |
| KLHL6_HUMAN | 0 | 0 | 0 | 0 | 1 | 0 | 0 | 0 | 0 | 0 | 0 | 0 | 1 | 7 | 1 | 1 | 48 | 0 | 0 | 2 |
| KLHL6_MOUSE | 0 | 0 | 0 | 0 | 1 | 0 | 0 | 0 | 0 | 0 | 0 | 0 | 1 | 7 | 1 | 1 | 51 | 0 | 0 | 1 |
| KLHL7_RAT | 0 | 0 | 0 | 0 | 1 | 0 | 0 | 0 | 0 | 0 | 0 | 0 | 1 | 7 | 1 | 1 | 48 | 0 | 0 | 0 |
| PON2_BOVIN | 0 | 0 | 0 | 0 | 0 | 1 | 0 | 0 | 0 | 0 | 0 | 0 | 1 | 6 | 1 | 1 | 55 | 0 | 0 | 0 |
| PON2_CANFA | 0 | 0 | 0 | 0 | 0 | 1 | 0 | 0 | 0 | 0 | 0 | 0 | 1 | 6 | 1 | 1 | 58 | 0 | 0 | 0 |
| PON2_HUMAN | 0 | 0 | 0 | 0 | 0 | 1 | 0 | 0 | 0 | 0 | 0 | 0 | 1 | 6 | 1 | 1 | 55 | 0 | 0 | 0 |
| PON2_RAT | 0 | 0 | 0 | 0 | 0 | 1 | 0 | 0 | 0 | 0 | 0 | 0 | 1 | 6 | 1 | 1 | 56 | 0 | 0 | 0 |
| PON3_MOUSE | 0 | 0 | 0 | 0 | 0 | 1 | 0 | 0 | 0 | 0 | 0 | 0 | 1 | 6 | 1 | 1 | 55 | 0 | 0 | 0 |
| PON3_RAT | 0 | 0 | 0 | 0 | 0 | 1 | 0 | 0 | 0 | 0 | 0 | 0 | 1 | 6 | 1 | 1 | 55 | 0 | 0 | 0 |
| VPS39_MOUSE | 0 | 0 | 0 | 0 | 0 | 0 | 0 | 0 | 0 | 0 | 0 | 1 | 1 | 6 | 1 | 1 | 52 | 0 | 0 | 1 |
| WBS16_MOUSE | 0 | 0 | 0 | 0 | 0 | 0 | 1 | 0 | 0 | 0 | 0 | 0 | 1 | 7 | 1 | 1 | 49 | 0 | 1 | 0 |
| AEE19_ARATH | 0 | 0 | 0 | 0 | 0 | 0 | 0 | 1 | 0 | 1 | 0 | 0 | 2 | 8 | 1 | 1 | 59 | 0 | 0 | 1 |
| BAMB_VIBF1 | 0 | 0 | 0 | 0 | 0 | 0 | 0 | 1 | 0 | 1 | 0 | 0 | 2 | 8 | 1 | 1 | 53 | 0 | 0 | 0 |

^*^WDSP_p represents the number of WD40 protein. WDSP_d represents the number of WD40 domain.
