# Supplementary material for: A Method for WD40 Repeat Detection and Secondary Structure Prediction
Source: PLoS One. 2013 Jun 11;8(6):e65705. doi: 10.1371/journal.pone.0065705 (PMC3679165; doi:10.1371/journal.pone.0065705)
Supplement: Table S7 — 68 proteins are unable to be identified by WDSP. (DOCX) [file pone.0065705.s011.docx]

**Table S7**. 68 proteins are unable to be identified by WDSP. 16 of them are due to incompleteness of repeats in the domain.

| reasons | ID | other methods^*^ | | | | | | | | | | | | WDSP^*^ | | | | | | |
| --- | --- | --- | --- | --- | --- | --- | --- | --- | --- | --- | --- | --- | --- | --- | --- | --- | --- | --- | --- | --- |
|  |  | Uniprot | Uniprot_d | Uniprot_p | SMART | SMART_d | SMART_p | PFAM | PFAM_d | PFAM_p | PROSITE | PROSITE_d | PROSITE_p | WDSP | WDSP_d | WDSP_p | avg. score | pentad | tetrad | traid |
| **too few repeats** | ATG18_PICPA | 2 | 1 | 1 | 2 | 1 | 1 | 1 | 1 | 1 | 0 | 0 | 0 | 5 | 1 | 1 | 68 | 0 | 0 | 0 |
|  | YAG1_SCHPO | 4 | 1 | 1 | 3 | 1 | 1 | 0 | 0 | 0 | 0 | 0 | 0 | 5 | 1 | 1 | 74 | 0 | 0 | 0 |
|  | DCAF6_PONAB | 7 | 1 | 1 | 7 | 1 | 1 | 4 | 1 | 1 | 0 | 0 | 0 | 5 | 1 | 1 | 84 | 0 | 1 | 2 |
|  | HSV2_EMENI | 2 | 1 | 1 | 2 | 1 | 1 | 2 | 1 | 1 | 0 | 0 | 0 | 5 | 1 | 1 | 64 | 0 | 0 | 0 |
|  | DCAF6_MOUSE | 7 | 1 | 1 | 7 | 1 | 1 | 4 | 1 | 1 | 0 | 0 | 0 | 5 | 1 | 1 | 84 | 0 | 1 | 2 |
|  | WDR25_HUMAN | 7 | 1 | 1 | 6 | 1 | 1 | 4 | 1 | 1 | 2 | 1 | 1 | 5 | 1 | 1 | 70 | 1 | 1 | 1 |
|  | DCAF6_HUMAN | 7 | 1 | 1 | 7 | 1 | 1 | 4 | 1 | 1 | 0 | 0 | 0 | 5 | 1 | 1 | 84 | 0 | 1 | 2 |
|  | HSV2_NEUCR | 2 | 1 | 1 | 2 | 1 | 1 | 2 | 1 | 1 | 0 | 0 | 0 | 5 | 1 | 1 | 54 | 0 | 0 | 1 |
|  | ATG18_CHAGB | 3 | 1 | 1 | 2 | 1 | 1 | 1 | 1 | 1 | 0 | 0 | 0 | 5 | 1 | 1 | 63 | 0 | 0 | 0 |
|  | RFWD3_AILME | 3 | 1 | 1 | 2 | 1 | 1 | 0 | 0 | 0 | 0 | 0 | 0 | 5 | 1 | 1 | 52 | 0 | 0 | 1 |
|  | WDR25_MOUSE | 7 | 1 | 1 | 5 | 1 | 1 | 3 | 1 | 1 | 2 | 1 | 1 | 5 | 1 | 1 | 72 | 1 | 0 | 2 |
|  | SEC31_PHANO | 6 | 1 | 1 | 4 | 1 | 1 | 2 | 1 | 1 | 1 | 1 | 1 | 4 | 1 | 1 | 73 | 0 | 1 | 0 |
|  | AMRA1_MOUSE | 3 | 1 | 1 | 3 | 1 | 1 | 1 | 1 | 1 | 1 | 1 | 1 | 4 | 1 | 1 | 76 | 0 | 1 | 0 |
|  | RFWD3_HUMAN | 3 | 1 | 1 | 3 | 1 | 1 | 0 | 0 | 0 | 0 | 0 | 0 | 4 | 1 | 1 | 58 | 0 | 0 | 1 |
|  | APC4_ARATH | 3 | 1 | 1 | 2 | 1 | 1 | 1 | 1 | 1 | 0 | 0 | 0 | 4 | 1 | 1 | 51 | 0 | 0 | 0 |
|  | AMRA1_HUMAN | 3 | 1 | 1 | 3 | 1 | 1 | 1 | 1 | 1 | 1 | 1 | 1 | 4 | 1 | 1 | 76 | 0 | 1 | 0 |
| **low score** | APC4_DICDI | 0 | 0 | 0 | 1 | 1 | 1 | 1 | 1 | 1 | 0 | 0 | 0 | 6 | 1 | 1 | 14 | 0 | 0 | 0 |
|  | APC4_SCHPO | 1 | 1 | 1 | 1 | 1 | 1 | 1 | 1 | 1 | 0 | 0 | 0 | 6 | 1 | 1 | 28 | 0 | 0 | 1 |
|  | APC4_YEAST | 0 | 0 | 0 | 0 | 0 | 0 | 1 | 1 | 1 | 0 | 0 | 0 | 7 | 1 | 1 | 29 | 0 | 0 | 0 |
|  | TAF1C_RAT | 0 | 0 | 0 | 1 | 1 | 1 | 0 | 0 | 0 | 0 | 0 | 0 | 6 | 1 | 1 | 31 | 0 | 0 | 0 |
|  | MED16_DICDI | 2 | 1 | 1 | 3 | 1 | 1 | 0 | 0 | 0 | 0 | 0 | 0 | 7 | 1 | 1 | 34 | 0 | 0 | 1 |
|  | APC4_HUMAN | 0 | 0 | 0 | 0 | 0 | 0 | 1 | 1 | 1 | 0 | 0 | 0 | 7 | 1 | 1 | 35 | 0 | 0 | 0 |
|  | APC4_MOUSE | 0 | 0 | 0 | 0 | 0 | 0 | 1 | 1 | 1 | 0 | 0 | 0 | 7 | 1 | 1 | 36 | 0 | 0 | 0 |
|  | WDR93_HUMAN | 1 | 1 | 1 | 0 | 0 | 0 | 0 | 0 | 0 | 0 | 0 | 0 | 7 | 1 | 1 | 36 | 0 | 0 | 0 |
|  | EIF3B_PICST | 7 | 1 | 1 | 0 | 0 | 0 | 0 | 0 | 0 | 0 | 0 | 0 | 7 | 1 | 1 | 37 | 0 | 0 | 1 |
|  | RIC1_HUMAN | 2 | 1 | 1 | 0 | 0 | 0 | 0 | 0 | 0 | 0 | 0 | 0 | 13 | 2 | 1 | 37 | 0 | 0 | 0 |
|  | RIC1_CAEEL | 3 | 1 | 1 | 0 | 0 | 0 | 0 | 0 | 0 | 0 | 0 | 0 | 14 | 2 | 1 | 37 | 0 | 0 | 3 |
|  | WDR93_MOUSE | 1 | 1 | 1 | 0 | 0 | 0 | 0 | 0 | 0 | 0 | 0 | 0 | 11 | 2 | 1 | 39 | 0 | 0 | 0 |
|  | RIC1_MOUSE | 2 | 1 | 1 | 0 | 0 | 0 | 0 | 0 | 0 | 0 | 0 | 0 | 12 | 2 | 1 | 40 | 0 | 0 | 1 |
|  | ATG21_YEAST | 3 | 1 | 1 | 3 | 1 | 1 | 1 | 1 | 1 | 0 | 0 | 0 | 7 | 1 | 1 | 41 | 0 | 0 | 1 |
|  | ATG21_YEAS7 | 5 | 1 | 1 | 3 | 1 | 1 | 1 | 1 | 1 | 0 | 0 | 0 | 7 | 1 | 1 | 41 | 0 | 0 | 1 |
|  | L2GL_DROME | 14 | 2 | 1 | 4 | 1 | 1 | 0 | 0 | 0 | 0 | 0 | 0 | 15 | 2 | 1 | 41 | 0 | 0 | 0 |
|  | DCAF4_BOVIN | 2 | 1 | 1 | 2 | 1 | 1 | 0 | 0 | 0 | 1 | 1 | 1 | 7 | 1 | 1 | 41 | 0 | 0 | 0 |
|  | ATG18_KLULA | 2 | 1 | 1 | 2 | 1 | 1 | 2 | 1 | 1 | 0 | 0 | 0 | 7 | 1 | 1 | 41 | 0 | 0 | 0 |
|  | APC4_PONAB | 0 | 0 | 0 | 0 | 0 | 0 | 1 | 1 | 1 | 0 | 0 | 0 | 6 | 1 | 1 | 41 | 0 | 0 | 0 |
|  | YBPD_SCHPO | 4 | 1 | 1 | 0 | 0 | 0 | 0 | 0 | 0 | 0 | 0 | 0 | 14 | 2 | 1 | 41 | 0 | 0 | 1 |
|  | EIF3B_CANAL | 7 | 1 | 1 | 0 | 0 | 0 | 0 | 0 | 0 | 0 | 0 | 0 | 7 | 1 | 1 | 41 | 0 | 0 | 1 |
|  | EIF3B_KLULA | 4 | 1 | 1 | 0 | 0 | 0 | 0 | 0 | 0 | 0 | 0 | 0 | 7 | 1 | 1 | 42 | 0 | 0 | 1 |
|  | HSV2_CANAL | 3 | 1 | 1 | 3 | 1 | 1 | 1 | 1 | 1 | 0 | 0 | 0 | 7 | 1 | 1 | 42 | 0 | 0 | 0 |
|  | MED16_EMENI | 0 | 0 | 0 | 2 | 1 | 1 | 0 | 0 | 0 | 0 | 0 | 0 | 8 | 1 | 1 | 42 | 0 | 0 | 0 |
|  | RIC1_DROME | 5 | 1 | 1 | 3 | 1 | 1 | 0 | 0 | 0 | 0 | 0 | 0 | 13 | 2 | 1 | 43 | 0 | 0 | 0 |
|  | TRI1_STRCO | 0 | 0 | 0 | 1 | 1 | 1 | 0 | 0 | 0 | 0 | 0 | 0 | 9 | 2 | 1 | 43 | 0 | 0 | 1 |
|  | EIF3B_CAEBR | 7 | 1 | 1 | 0 | 0 | 0 | 0 | 0 | 0 | 0 | 0 | 0 | 10 | 2 | 1 | 43 | 0 | 0 | 0 |
|  | EIF3B_MAGO7 | 7 | 1 | 1 | 0 | 0 | 0 | 0 | 0 | 0 | 0 | 0 | 0 | 7 | 1 | 1 | 43 | 0 | 0 | 0 |
|  | CB044_DANRE | 2 | 1 | 1 | 0 | 0 | 0 | 0 | 0 | 0 | 0 | 0 | 0 | 7 | 1 | 1 | 43 | 0 | 0 | 0 |
|  | VPS41_YEAST | 5 | 1 | 1 | 2 | 1 | 1 | 1 | 1 | 1 | 0 | 0 | 0 | 7 | 1 | 1 | 44 | 0 | 0 | 1 |
|  | BCAS3_DROME | 3 | 1 | 1 | 0 | 0 | 0 | 0 | 0 | 0 | 0 | 0 | 0 | 7 | 1 | 1 | 44 | 0 | 0 | 0 |
|  | EIF2A_DICDI | 4 | 1 | 1 | 0 | 0 | 0 | 0 | 0 | 0 | 0 | 0 | 0 | 7 | 1 | 1 | 44 | 0 | 0 | 0 |
|  | TOLB_HAEI8 | 0 | 0 | 0 | 1 | 1 | 1 | 0 | 0 | 0 | 0 | 0 | 0 | 6 | 1 | 1 | 44 | 0 | 0 | 0 |
|  | FRITZ_HUMAN | 2 | 1 | 1 | 0 | 0 | 0 | 0 | 0 | 0 | 0 | 0 | 0 | 7 | 1 | 1 | 44 | 0 | 0 | 0 |
|  | TOLB_PASMU | 0 | 0 | 0 | 2 | 1 | 1 | 0 | 0 | 0 | 0 | 0 | 0 | 6 | 1 | 1 | 44 | 0 | 0 | 0 |
|  | TOLB_HAEIN | 0 | 0 | 0 | 1 | 1 | 1 | 0 | 0 | 0 | 0 | 0 | 0 | 6 | 1 | 1 | 45 | 0 | 0 | 0 |
|  | EMB30_CAEEL | 2 | 1 | 1 | 0 | 0 | 0 | 1 | 1 | 1 | 0 | 0 | 0 | 9 | 2 | 1 | 45 | 0 | 0 | 1 |
|  | TOLB_HAEIG | 0 | 0 | 0 | 1 | 1 | 1 | 0 | 0 | 0 | 0 | 0 | 0 | 6 | 1 | 1 | 45 | 0 | 0 | 0 |
|  | L2GL_DROPS | 14 | 2 | 1 | 5 | 1 | 1 | 0 | 0 | 0 | 0 | 0 | 0 | 14 | 2 | 1 | 45 | 0 | 0 | 0 |
|  | TOLB_HAES1 | 0 | 0 | 0 | 2 | 1 | 1 | 0 | 0 | 0 | 0 | 0 | 0 | 6 | 1 | 1 | 46 | 0 | 0 | 0 |
|  | EIF3B_YEAST | 6 | 1 | 1 | 0 | 0 | 0 | 0 | 0 | 0 | 0 | 0 | 0 | 7 | 1 | 1 | 46 | 0 | 0 | 0 |
|  | EIF3B_CAEEL | 6 | 1 | 1 | 0 | 0 | 0 | 0 | 0 | 0 | 0 | 0 | 0 | 10 | 2 | 1 | 46 | 0 | 0 | 0 |
|  | ATG21_CANAL | 2 | 1 | 1 | 2 | 1 | 1 | 2 | 1 | 1 | 0 | 0 | 0 | 7 | 1 | 1 | 46 | 0 | 0 | 0 |
|  | TOLB_HAES2 | 0 | 0 | 0 | 2 | 1 | 1 | 0 | 0 | 0 | 0 | 0 | 0 | 6 | 1 | 1 | 46 | 0 | 0 | 0 |
|  | EIF3B_ASHGO | 10 | 2 | 1 | 0 | 0 | 0 | 0 | 0 | 0 | 0 | 0 | 0 | 9 | 2 | 1 | 46 | 0 | 0 | 1 |
|  | HIR2_ASHGO | 8 | 1 | 1 | 2 | 1 | 1 | 0 | 0 | 0 | 0 | 0 | 0 | 11 | 2 | 1 | 46 | 0 | 0 | 0 |
|  | MED16_AEDAE | 3 | 1 | 1 | 0 | 0 | 0 | 0 | 0 | 0 | 0 | 0 | 0 | 10 | 2 | 1 | 46 | 0 | 0 | 1 |
|  | MED16_DROME | 3 | 1 | 1 | 0 | 0 | 0 | 0 | 0 | 0 | 0 | 0 | 0 | 9 | 2 | 1 | 47 | 0 | 0 | 0 |
|  | FBW1_ARATH | 2 | 1 | 1 | 0 | 0 | 0 | 0 | 0 | 0 | 0 | 0 | 0 | 6 | 1 | 1 | 47 | 0 | 0 | 0 |
|  | EIF3B_NEUCR | 5 | 1 | 1 | 0 | 0 | 0 | 0 | 0 | 0 | 0 | 0 | 0 | 9 | 2 | 1 | 47 | 0 | 0 | 0 |
|  | 2ABA_ORYSJ | 6 | 1 | 1 | 6 | 1 | 1 | 0 | 0 | 0 | 0 | 0 | 0 | 7 | 1 | 1 | 47 | 0 | 0 | 2 |
|  | EIF3B_VANPO | 7 | 1 | 1 | 0 | 0 | 0 | 0 | 0 | 0 | 0 | 0 | 0 | 8 | 1 | 1 | 47 | 0 | 0 | 1 |

^*^similar with **Table S6**
